# Two-Tiered Control of Epithelial Growth and Autophagy by the Insulin Receptor and the Ret-Like Receptor, Stitcher

**DOI:** 10.1371/journal.pbio.1001612

**Published:** 2013-07-23

**Authors:** Fergal O'Farrell, Shenqiu Wang, Nadja Katheder, Tor Erik Rusten, Christos Samakovlis

**Affiliations:** 1 Department of Molecular Biosciences, The Wenner-Gren Institute, Stockholm University, Stockholm, Sweden; 2 Institute for Cancer Research, Oslo University Hospital, Montebello, Oslo, Norway; 3 Centre for Cancer Biomedicine, Oslo University Hospital, Montebello, Oslo, Norway; 4 Cancer Biology and Genetics Program, Memorial Sloan-Kettering Cancer Center, New York, New York, United States of America; Stanford University School of Medicine, Howard Hughes Medical Institute, United States of America

## Abstract

Body size in *Drosophila* larvae, like in other animals, is controlled by nutrition. Nutrient restriction leads to catabolic responses in the majority of tissues, but the *Drosophila* mitotic imaginal discs continue growing. The nature of these differential control mechanisms that spare distinct tissues from starvation are poorly understood. Here, we reveal that the Ret-like receptor tyrosine kinase (RTK), Stitcher (Stit), is required for cell growth and proliferation through the PI3K-I/TORC1 pathway in the *Drosophila* wing disc. Both Stit and insulin receptor (InR) signaling activate PI3K-I and drive cellular proliferation and tissue growth. However, whereas optimal growth requires signaling from both InR and Stit, catabolic changes manifested by autophagy only occur when both signaling pathways are compromised. The combined activities of Stit and InR in ectodermal epithelial tissues provide an RTK-mediated, two-tiered reaction threshold to varying nutritional conditions that promote epithelial organ growth even at low levels of InR signaling.

## Abstract 2

The *Drosophila* Ret-like receptor, Stit, upholds signaling from the protein complex TORC1 during wing epithelial development, promoting growth under normal conditions and protecting tissues from an anabolic to catabolic switch in response to starvation.

## Author Summary

Growth of organs, or anabolism, is tightly controlled by nutritional and hormonal cues such as insulin-like peptides that also suppress autophagy through their receptors and downstream growth pathway. Starvation conditions induce growth arrest and catabolism (involving autophagy) in some tissues while sparing the growth of other prioritized organs. The mechanism behind this tissue-specific regulation of growth versus catabolism is largely unknown. In this study, we show that Stitcher, a *Drosophila* Ret-oncogene-like growth factor receptor, controls epithelial tissue growth. Stitcher, working in parallel with the Insulin receptor, endows epithelial organs, such as imaginal wing discs, with resistance to low nutrient and insulin conditions by suppressing autophagy and, at the same time, promotes cell division and growth in these tissues. Thus, Stitcher and the Insulin receptor work together to allow a two-threshold response to starvation in epithelial tissues. In cancer, this pathway is almost invariably constitutively stimulated, and so we postulate that oncogenic mutations of Ret promote tumor growth partly by counteracting the tumor suppressive effects of autophagy.

## Introduction

Cellular and organ growth (anabolism) in animals is regulated by complex interactions of nutritional and hormonal cues. As accumulation of cell mass usually precedes cell division, cellular growth is intimately coupled to proliferation and net organ growth. In all eukaryotes studied, the evolutionarily conserved protein complex TORC1 (target of rapamycin complex 1) integrates nutritional and hormonal cues and translates this information into cellular growth and proliferation. When sufficient ATP and amino acids are present, TOR kinase directly phosphorylates S6K and 4E-BP and stabilizes Myc to promote the activity of the protein translation machinery, thereby permitting protein production and cell growth [1],[2]. The amino-acid-sensing machinery is located on a late endosomal compartment, where a small GTPase heterodimer consisting of RagA/B/GTR1 and RagC/D/GTR2, together with Rheb, is required to stimulate TORC1 activity upon amino acid stimulation [3]–[5].

In animals, complex hormonal regulation is layered upon the permissive cellular nutrient sensing to ensure coordinated tissue growth. Results from genetically tractable models revealed that the Insulin/Insulin Growth Factor (IGF) ligands and receptors are the principal organ growth regulators coupled to nutrition [1],[6],[7]. *Drosophila* Insulin-like peptides (dILPs) signal through an evolutionarily conserved growth promoting pathway initiated by binding of the adaptor proteins, Chico and Lnk, to the intracellular domain of the InR [8],[9]. Subsequent recruitment of the Phosphatidylinositol-kinase class I (PI3K-I) leads to recruitment of Akt and PDK1 kinases to the plasma membrane through their respective phosphatidylinositol 3,4,5 trisphosphate (PIP3)-lipid binding pleckstrin homology (PH) domains. PDK1 phosphorylates and activates Akt [10]–[12], which in turn activates TORC1 by inhibiting its negative regulators PRAS40 and TSC1/TSC2 [13]–[16].

Suboptimal nutrient conditions during both animal development and homeostasis can be compensated for by the differential control of growth and catabolism in different organs. The molecular mechanisms underlying these tissue-specific responses *in vivo* are only beginning to be elucidated. Shortage of amino acids is sensed by the larval fat body resulting in the activation of autophagy and of an unknown relay signal reducing systemic dILP levels and growth [1],[17]–[20].

Prolonged starvation halts the growth of most polyploid larval tissues including the gut, fat body, and epidermis [21]. Strikingly, the imaginal tissues that will make up the adult fly during metamorphosis continue to grow even when amino acid levels in the hemolymph drop [21],[22]. A recent elegant study revealed that the continued cycling of neuroblasts in the *Drosophila* brain, when insulin signaling is low, is supported by growth signaling from the RTK, ALK (Anaplastic Lymphoma Kinase). ALK bypasses the TORC1 requirements for growth but still acts through the direct TORC1 targets, 4E-BP and S6K [22],[23].

Stitcher (Stit) is a Ret-like, receptor tyrosine kinase (RTK) activated upon epidermal wounding in *Drosophila* embryos. Stit signaling induces the transcriptional activation of genes involved in barrier repair and the coordinated cytoskeletal rearrangements leading to epidermal wound closure [24]. Here, we report that Stit also promotes growth in the *Drosophila* epithelial imaginal wing discs. We show that in these organs, Stit induces growth in parallel to InR, but also suppresses autophagy upon low InR signaling through the canonical PI3K-I/TORC1 pathway. Thus, the Stit and InR RTKs ensure a two-threshold response to TORC1 activity in proliferating epithelial tissues, increasing their repertoire of reactions to nutrient stress.

## Results

### Stit Is Required for Wing Growth

**Figure 1:**
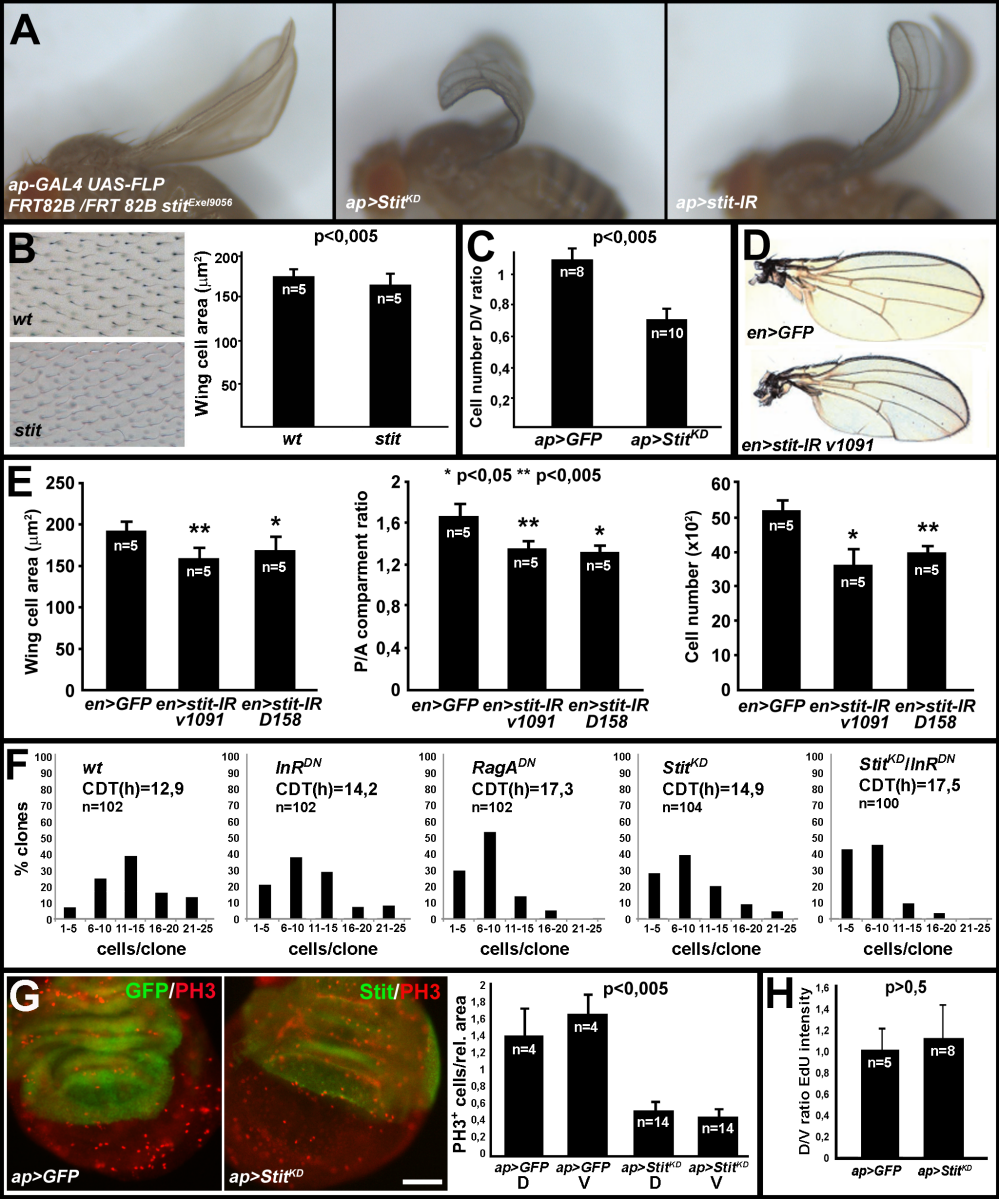
Stit is required for optimal growth of the wing epithelium. (A) Stit inactivation in the dorsal wing compartment using *ap*-*GAL4*-induced FRT *stit* mutant clones or expression of either *Stit^KD^* or *stit-IR* caused an upwardly bent adult wing. (B) Wing cell area decreased in *stit* mutant clones compared to wild-type clones. (C) The expression of *Stit^KD^* in the dorsal wing compartment led to a 33% reduction of the dorsal/ventral (D/V) cell number ratio (>15,000 cells from 10 *Stit^KD^* pupal wings counted) relative to *GFP* expressing controls (8 animals, >15,000 cells counted). Student's *t* test, *p*<0.005. (D) Expression of *stit-IR* in the posterior compartment (*en-GAL4*) caused a backwardly bent wing. (E) The posterior compartment of *en>stit-IR* wings was reduced in size due to a reduction in both the total cell number and wing cell area relative to GFP-expressing control wings. (F) *hs-flp*;*actin*>*CD2,stop*>*GAL4* (AFG4)-generated clones revealed a general reduction of proliferation and an increase in cell doubling time (CDT) when insulin (*InR^DN^*) or amino acid signaling (*RagA^DN^*) was reduced relative to clones expressing GFP alone (48 h after heatshock). A similar effect was observed in *Stit^KD^*-expressing clones, whereas simultaneous reduction of Stit and InR signaling led to a further reduction in proliferation. The number of clones examined (*n*) for each genotype is indicated. (G) Analysis of Phospho-histone 3 (PH3)-positive mitotic profiles in third instar larval wing discs revealed a general (dorsal, **D**, and ventral, **V**, in graph) reduction in the number of mitotically active cells within *ap*>*Stit^KD^* wing discs compared to control, Student's *t* test, *p*<0.005 when comparing either control compartment with either *Stit^KD^* compartment. The number of discs examined (*n*) is indicated. Scale bar, 50 µm. (H) 10 min of EdU incorporation did not show a compartment-specific change in cells entering into S-phase in *ap*>*Stit^KD^* discs versus *ap>GFP* control discs. However, the overall (both dorsal and ventral) labeling was more sparse in *Stit^KD^* wings, although the D/V ratio was close to 1. The number of wing/discs examined (*n*) is indicated. All error bars indicate standard deviation. The nonautonomous compensatory growth effect is explored further in Figure S1.

**Figure 2:**
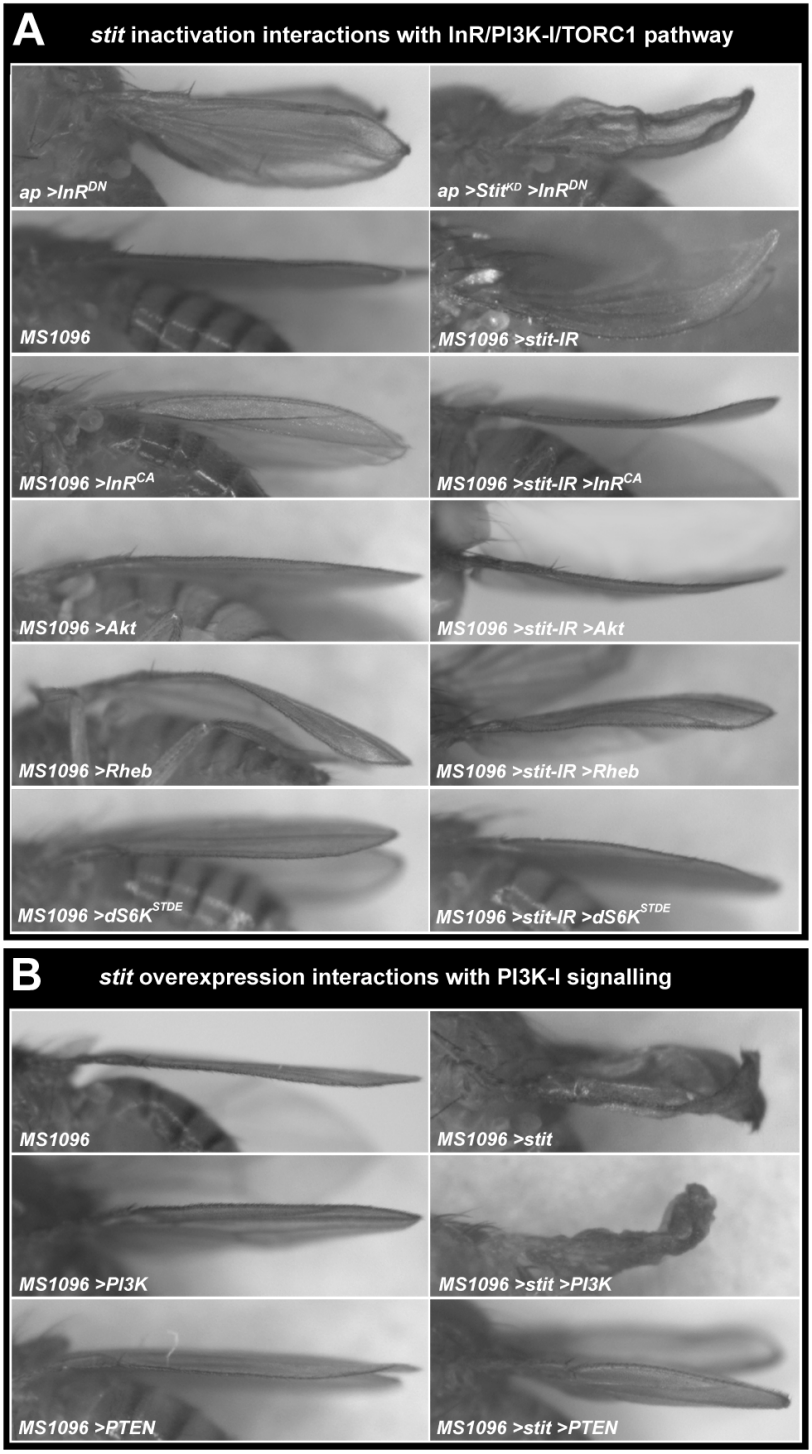
Genetic interactions of *stit* with mutations affecting the PI3K-I/TORC1 pathway. (A) InR receptor inactivation in the dorsal compartment (*ap>InR^DN^*) reduced total wing size and caused a minor wing bending primarily around the margin. Co-reduction of Stit activity (by *Stit^KD^*) further reduced wing size and gave an upwardly bent wing in addition to the bending at the margin. The *stit-IR* wing bending phenotypes could be suppressed by co-expression of a constitutively active form of the InR (*InR^CA^*), *Rheb*, *Akt*, and constitutively active *dS6K* (*dS6K^STQE^*), indicating that *stit* acts via or in parallel to the PI3K-I/TORC1 cassette. Dorsal expression of Rheb led to a downwardly bent wing, indicative of overgrowth. This phenotype was also observed upon mild expression of wild-type *stit* (see Figure S2). (B) Overexpression of *stit* by *MS1096*-*GAL4* led to a severe crumpled wing phenotype. This could be further enhanced by co-expression of *PI3K*-*I* or suppressed by co-expression of *PTEN*. See also Table S1.

*stit* mutants die as pupae with melanized abdomens, and Stit protein was detected in several developing imaginal epithelial tissues (the wing, thorax, leg discs, and abdominal histoblasts), but was absent in eye-antennal disc (Figure S1A,B and unpublished data) [24]. To uncover a potential Stit function in epithelial tissue development, we focused on the wing imaginal discs. The larval wing discs generate a dorsal and a ventral epithelial cell layer, which appose each other during pupation to form the adult wing blade. We first made FRT *stit^Exel9056^* and *stit^Ex266^* mutant clones in the future dorsal surface of the wing using *apterous* (*ap*)-*GAL4* to drive *UAS-FLPase*. This resulted in strong upwardly bent wings with no apparent defects in vein patterning or hair orientation. This phenotype suggested that Stit controls the balanced growth of the dorsal and ventral wing compartments (Figure 1A). As *stit* mutant cells showed residual immunoreactivity during larval stages (Figure S1A), we additionally used a transgene encoding a kinase defective variant of Stit (*Stit^KD^*) to acutely disrupt endogenous Stit as well as *stit* RNAi-expressing trangenes (*stit-IR*). Broadly expressed *Stit^KD^* by *daughterless (da)*-*GAL4* caused pupal lethality resembling the *stit* mutant phenotype. Expression of *Stit^KD^* or *stit*-*IR* in the dorsal portion of the wing using either *ap*-GAL4 or *MS1096*-GAL4 reproducibly caused upwardly bent wings, but the bending was more severe than that caused by the mutant clones (Figures 1A and 2A). Similarly, expression of *Stit^KD^* and *stit*-*IR* using *engrailed* (*en*)-*GAL4* resulted in bending of the posterior wing part, suggesting that Stit coordinates shape and growth in the entire tissue (Figure 1D and not shown). Overexpression of wild-type *stit* (*ap>stit*) could rescue the wing phenotypes caused by either *Stit^KD^* or *stit*-*IR* (unpublished data), suggesting that both *Stit^KD^* and *stit*-*IR* can act as potent and specific inhibitors of Stit function. In conclusion, clones of *stit* cells and compartmentalized *stit* inactivation in the wing resulted in growth defects leading to tissue shape alterations.

### Stit Is Required for Cellular Proliferation During Tissue Growth

To determine the cellular defects leading to wing shape changes upon Stit disruption, we first analyzed *stit* mutant clones in adult wings. We generated wings composed of *forked* (*f*)-marked wild-type cells or *f*-marked *stit* mutant cells opposing unmarked minute cells to assess the growth and competitiveness of *stit* mutant cells. In this setting, wild type almost completely outgrew the minute clones. We found a small, but significant reduction in both the size (wing cell area) and total number of cells in *stit* clones compared to wild-type clones (Figures 1B and S1C). The overall size of wings containing *stit* mutant clones was also slightly reduced compared to wings composed of *f*-marked wild type cells. Similarly, *stit* inactivation by expression of *stit*-*IR^v1091^* or *stit-IR^D158^* in the posterior compartment by *en*-*GAL4* resulted in a 20%–30% reduction in cell numbers, a significant cell size reduction and an overall size reduction of the posterior wing compartment (Figure 1D,E). This indicated that Stit controls cell growth and/or proliferation in the wing imaginal discs. To directly assess imaginal epithelial growth, we co-expressed *GFP* with *Stit^KD^* or *stit*-*IR* in the dorsal compartment using *ap-GAL4*. We labeled discs with anti-DE-Cadherin and determined cell numbers and their ratios in the juxtaposed dorsal and ventral epithelial layers at 20–40 h after pupal formation (APF) (Figures 1C and S1D). Wing discs from *ap*>*GFP* control pupae had a D/V cell ratio close to the expected value 1. *ap*>*Stit^KD^*, however, showed a D/V cell ratio of 0.67, indicating a 33% reduction of cells within the dorsal compartment compared to the ventral or compared to the wild-type control ratio. The shape of pupal wing cells expressing *Stit^KD^* was different than control wing cells. They were shorter than their ventral counterparts, or conversely their ventral counterparts appeared taller (Figure S1D, X–Z section). This D/V cell shape difference was not observed in the control and suggests a compensatory mechanism, where either the dorsal cells stretch to cover the larger ventral area or conversely the ventral cells compact to accommodate a reduced dorsal surface. Thus, *stit* mutant clones or localized expression of *Stit^KD^* and *stit-IR* caused a decrease in cell numbers at pupal stages and a reduction in both cell size and number in the adult wing. We graded the impact of the genetic manipulations to cell numbers in the order: *Stit^KD^*>*stit*-*IR*>*stit* null mutant. This suggested a prominent Stit protein perdurance in the null mutant clones in the wing and prompted us to focus our analysis on the phenotypes generated by the *stit* RNAi and the dominant negative transgenes.

We first assessed whether the reduction in cell numbers was due to an increase in cell death or a decrease in cell proliferation. Expression of *stit*-*IR* and *Stit^KD^* in the dorsal compartment of wing discs did not lead to increase in cell death as assessed by TUNEL labeling or Caspase 3 staining (Figure S1G). Moreover, concurrent expression of p35, a baculoviral caspase inhibitor, together with *stit-IR* or *Stit^KD^* did not ameliorate the wing bending caused by *stit* inactivation (unpublished data), suggesting that Stit does not influence apoptosis. To examine its role in cell proliferation, we inactivated *stit* in clones using *hs-flp;Act>CD2>GAL4* (AFG4) to drive *Stit^KD^* and *GFP* expression. In parallel, we used the same approach to express *InR^DN^* or *RagA^DN^* as positive controls. We determined the number of GFP positive cells in individual clones 48 h after clone induction. We plotted the percentage of clones within defined size intervals and calculated the mean cell doubling time (CDT) for each genotype. The cell numbers in GFP expressing control clones produced a bell-shaped distribution, where the majority (40%) of clones were comprised of 11–15 cells. This resulted in a CDT of 12.9 h (Figure 1F), in close agreement with previous findings [25]. The expression of *InR^DN^* or *RagA^DN^* shifted this bell-shaped curve to the left, indicating an increase in the frequency of clones comprised of fewer cells and a corresponding increase in mean CDT to 14.2 and 17.3 h, respectively. Similarly, *Stit^KD^* expression reduced the number of cells per clone, resulting in a mean CDT of 14.9 h, suggesting that Stit is required for epithelial cell proliferation.

To pinpoint a potential function of Stit in cell proliferation, we labeled *ap*>*Stit^KD^* or *ap>stit*-*IR* discs with anti-phosphohistone H3 (PH3), EdU (a BrdU analog), and anti-dGeminin. We observed a strong reduction in the labeling of all three markers in *ap*>*Stit^KD^* or *ap>stit-IR* discs compared to the control discs (Figures 1G, 1H, S1E and unpublished data). Surprisingly, the 3-fold decrease of anti-PH3 positive cells was not confined in the dorsal compartment but was evident in the entire wing pouch of *ap*>*Stit^KD^*. The EdU and Geminin stainings also showed a general reduction in labeling cells upon *stit* inactivation compared to control discs (Figure S1E). This generalized decrease of proliferative markers in the wing discs in response to localized Stit inactivation is in congruence with a nonautonomous mechanism that coordinates wing growth in response to local perturbations [26]. We labeled discs with the G2/M phase marker Cyclin B. Cyclin B expression is dynamic during the cell cycle, accumulating from the end of S-phase, through G2, abating during mitosis. *ap*>*Stit^KD^* expressing wing discs showed increased Cyclin B levels in the ventral compartment, suggesting a compensatory prolongation or an arrest in G2 therein (Figure S1F). Thus, apart from the global reduction of mitotic markers and the reduction of the wing size upon Stit inactivation, we did not detect any selective block in the cell cycle. Presumably, the decrease in dorsal proliferation occurs early and continuously during development, while the ventral compensation follows in response. We conclude that *stit* is required for normal levels of cell proliferation during wing development. Rather than playing a direct role controlling the cell cycle, Stit is more likely required for cellular growth.

### Stit and InR Function in Parallel to Promote Growth through TORC1

Inactivation of *stit* in the dorsal compartment either by *MS1096*>*stit*-*IR* or by *ap*>*Stit^KD^* generated strong upwardly bent wings resembling the defects caused by the disruption of the InR and other growth regulators (Figures 1A, 2A and Table S1) [27],[28]. *ap*-*GAL4*-driven *InR^DN^* caused a strong reduction in wing size and bending primarily around the margin (Figure 2A). This contrasted the bending of the entire wing blade generated by *ap*>*Stit^KD^* expression (Figure 1A). The difference in wing bending at the margin caused by *InR^DN^* versus the bending of the entire wing blade caused by *Stit^KD^* suggests a spatial control of Stit and InR activation in the discs. Co-expression of *Stit^KD^* and *InR^DN^* in the dorsal compartment further reduced wing size and increased the bending compared to the defects caused by *InR^DN^* alone (Figure 2A). These results suggest that Stit and InR function in parallel to control wing growth. To further examine the effect of inactivating both receptors on wing cell proliferation, we generated *hs-flp;Act>CD2>GAL4* clones expressing both *Stit^KD^* and *InR^DN^* and determined the number of cells per clone. The fraction of clones composed of fewer cells increased, leading to a marked increase in CDT (17.5 h) compared to the effect caused by inactivation of either receptor alone. This suggests that both Stit and InR are required for optimal tissue growth (Figure 1F). We therefore examined potential genetic interactions of *stit* with mutations affecting the PI3K-I/TORC1 signaling cassette. Overexpression of Stit by *MS1096>stit* causes severe crumpling of the whole blade (Figure 2B). Milder overexpression in the dorsal surface by *ap>stit* at 18°C caused a gentle bend of the wing downwards, indicative of an overgrowth of the dorsal surface (Figure S2A). A similar downward bending was generated by overexpression of the growth activator Rheb in the dorsal compartment using *MS1096>Rheb* (Figure 2A). The wing overgrowth phenotypes caused by the overexpression of Stit in the dorsal compartment were accompanied by an increase of BrdU and anti-PH3 in the dorsal compartment (Figure S2B). Thus, the changes in wing shape provide a sensitive assay for Stit function in tissue growth. We tested whether the defects caused by *stit* inactivation or overexpression can be modified by an array of loss-of-function alleles and overexpression constructs of genes regulating cell death, cell cycle control, and growth (Table S1). We did not detect any interactions with mutations affecting cell cycle progression. However, *MS1096*-*GAL4*-driven co-expression of *stit*-*IR* together with activating components of the PI3K-I/TORC1 pathway—PI3K-I, Akt, and Rheb—suppressed the *stit* bent wing phenotype (Figure 2A). Similarly, simultaneous inactivation of *stit* and *PTEN* (*PTEN-IR*) gave a flatter wing, suppressing the effect of *stit* knock down (Table S1). Furthermore, expression of an activated form of dS6K kinase (the *Drosophila* ortholog of p70 S6K, a direct TORC1 target) was sufficient to suppress the *stit* inactivation wing-bending phenotype, indicating that increased TORC1 signaling at any level of the intracellular pathway is sufficient to compensate for a lack of *stit*. Conversely, the wing phenotype resulting from *stit* overexpression was suppressed by expression of PTEN, a negative regulator of the pathway and could be enhanced by increasing levels of positive regulators or effectors of the TORC1 pathway, PI3K-I, Akt, Rheb, and dS6K (Figure 2B, Table S1). This implied that Stit activates either the PI3K-I pathway or a novel TORC1 regulatory pathway.

### Stit Drives the PI3K-I/TORC1 Pathway to Support Growth and Suppress Autophagy

**Figure 3:**
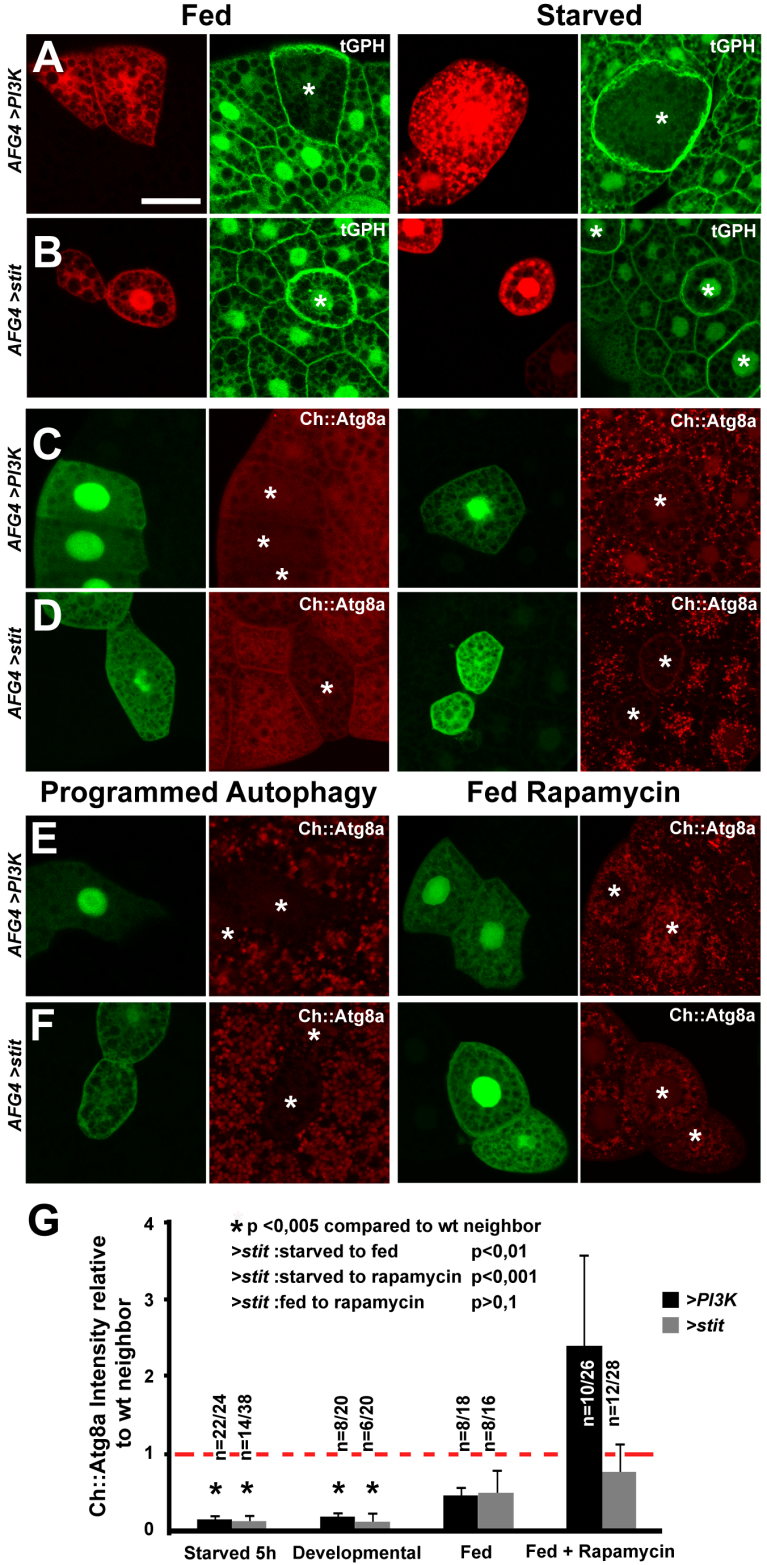
Stit activates PI3K-I to support growth and suppress starvation-induced autophagy. (A) Clonal overexpression in the fat body (marked by RFP, asterisk) of *PI3K-CaaX* led to increased recruitment of GFP-tagged PH probe (*tGPH*) to the fat body cell plasma membrane and cell enlargement. This was more evident under starvation conditions, where membrane-bound levels of GFP-PH declined in neighboring cells. (B) Clones expressing *stit* were slightly enlarged, more rounded, and had higher membrane-bound GFP-PH levels than neighbor cells. This persisted upon starvation. (C) Clonal overexpression of *PI3K-CaaX* or (D) *stit* (marked by GFP, asterisk) in the fat body of larvae expressing a Cherry-tagged Atg8a reporter expressed under the control of a fat body promoter showed that both PI3K-CaaX and Stit can block the starvation-induced punctate accumulation of Ch::Atg8a. Quantified in (G). (E) Clonal overexpression of either *PI3K-CaaX* (E) or *stit* (F) in larvae during programmed autophagy (P.A.) demonstrated that both can block Ch::Atg8a accumulation in the expressing cells. Quantified in (G). Feeding the TORC1 inhibitor rapamycin to larvae expressing *PI3K-CaaX* (E) or *stit* (F) in clones reverted the Stit-mediated block of Ch::Atg8a puncta accumulation. Quantified in (G). (G) The intensity of Ch::Atg8a in AFG4-positive cells was measured and compared to the nearest neighbor cells to calculate fold changes where a value of 1 (red hatched line) indicates no difference to the normal autophagy response to each condition (starved, fed, P.A./developmental, or rapamycin induced) observed in wild-type neighbor cells. Stit and PI3K-I could both suppress programmed and starvation-induced Ch::Atg8a accumulation/intensity increase. * indicates Student's *t* test scores of significance (*p*<0.005) between overexpressing cells and wild-type neighbor cells, while inset Student's *t* test scores indicate *p* values of the difference in response of *stit*-expressing cells/wild-type neighbor cells between conditions—that is, starved and fed. The number of transgene-expressing cells/wild-type neighbor cells where Ch::Atg8a intensity was measured (*n*) is indicated. Error bars indicate standard deviation. Scale bar, 50 µm. See also Figure S3.

To investigate the postulated role of Stit in cellular growth, we turned to the larval fat body, which is composed of endoreplicating cells growing without cellular division and thereby facilitates the analysis of cellular growth. Stit is not expressed in the fat body and its inactivation by *Stit^KD^* expression in this tissue did not lead to any discernible cell growth phenotype (Figure S3B,C). To investigate if Stit can induce PI3K-I activation and cellular growth, we overexpressed *stit* and *UAS-RFP* in clones in larvae expressing the *GFP*-*PH* (*tGPH*) reporter, which is recruited by PIP3 at the plasma membrane, thus reflecting PI3K-I activity. In parallel, we generated clones overexpressing a membrane targeted PI3K-I (PI3K-CaaX) and *Stit^KD^* as positive and negative controls (Figures 3 and S3). As expected, clonal overexpression of *PI3K*-*CaaX* caused a pronounced cell overgrowth and induced membrane accumulation of GFP, while *Stit^KD^* had no effects (Figures 3A and S3B,C). *stit* overexpressing cells showed an accumulation of the GFP-PH signal but not significant overgrowth compared to their wild-type neighbors (Figure 3B). Upon 24 h starvation, however, *PI3K*-*CaaX- or stit*-expressing cells were clearly larger and maintained a strong cortical GFP-PH signal compared to their neighbors (Figure 3A,B). This analysis suggests that Stit, like InR signaling, can activate PI3K-I and spare fat body cells from starvation-induced size reduction [19],[20]. To investigate the potential regulatory role of Stit in starvation-induced autophagy, a TORC1-regulated process, we assessed the accumulation of a Cherry-tagged Atg8a autophagy reporter in clones overexpressing Stit. A starvation period of 5 h induced a punctate Ch::Atg8a accumulation in wild-type cells. This increase in Ch::Atg8a was not evident in cells overexpressing either *stit* or PI3K-I (Figure 3C, 3D, and 3G). Similarly, the developmental wave of programmed autophagy (P.A.) observed in fed larvae just before pupation was blocked as efficiently by Stit as by PI3K-I (Figure 3E, 3F, and 3G) [20]. Thus, Stit overexpression can spare fat body cells from starvation-induced cell size reduction and autophagy. These observations implicate TORC1 as a downstream effector of Stit. To test this hypothesis we reared larvae overexpressing *stit* or *PI3K-CaaX* with 50 µM rapamycin (a potent inhibitor of TORC1 activity) and investigated their ability to block Ch::Atg8a accumulation. Rapamycin treatment for 24 h abolished the inhibitory effect of *stit* or *PI3K-I* on Ch::Atg8a accumulation upon starvation (Figure 3E, 3F, and 3G), thus indicating that Stit-mediated suppression of autophagy requires TORC1 activation.

**Figure 4:**
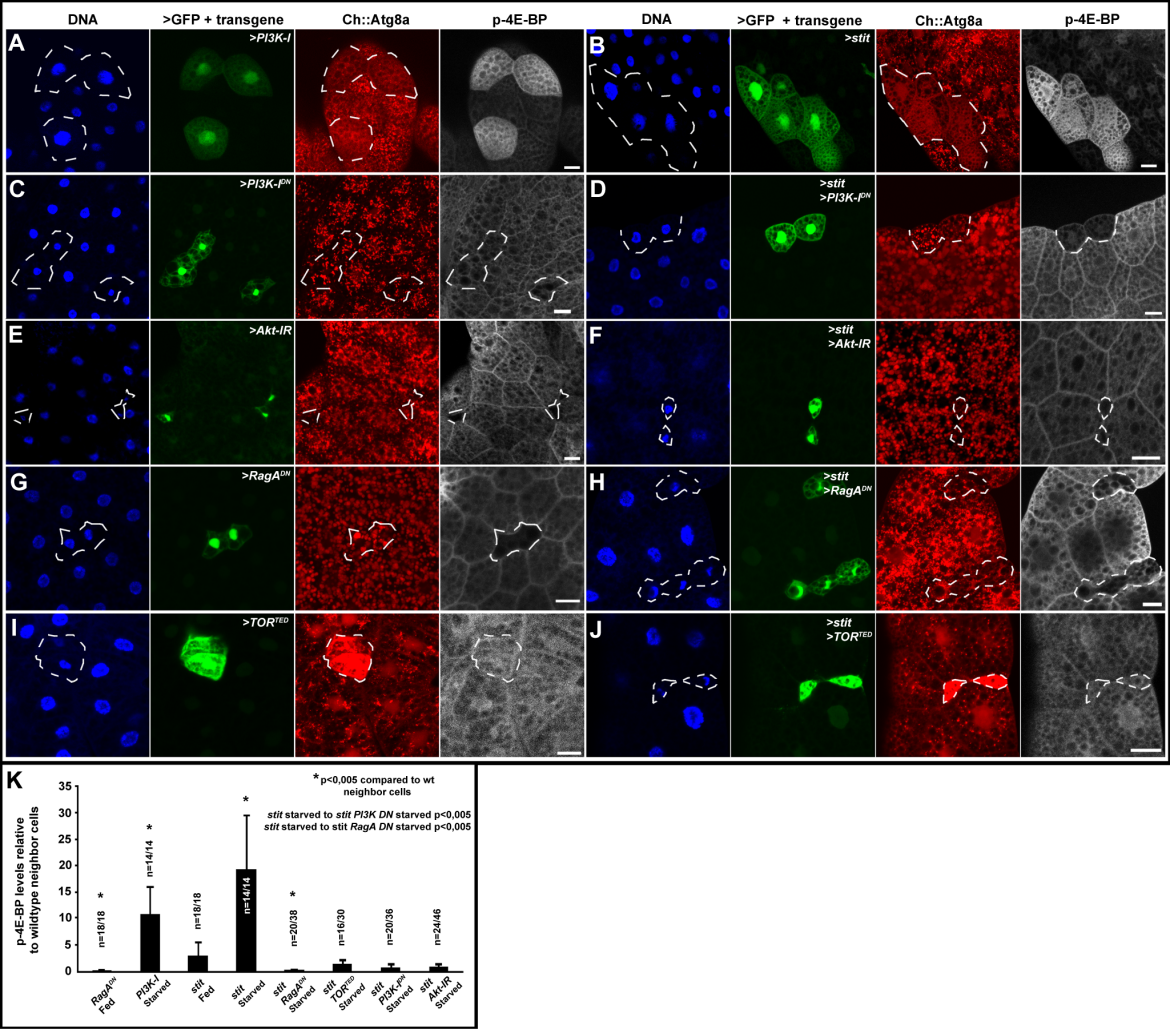
The PI3K-I/TORC1signalling cassette is required for Stit-dependent protection against starvation. Clonal expression (cells marked by GFP/hatched line) of *stit* and/or transgenes interfering with TORC1 signaling components in the fat body of starved (5 h) larvae expressing Ch::Atg8a under the control of a fat body promoter (A) *PI3K-I*-expressing cells have larger nuclei, are protected from starvation-induced Ch::Atg8a puncta formation, and maintain high p-4E-BP levels compared to wild-type neighbors. (B) *stit*-expressing cells behave like *PI3K-I*-expressing cells in (A). (C) *PI3K-I^DN^*-expressing cells are smaller and show Ch::Atg8a autophagic puncta and low levels of p-4E-BP (indistinguishable from neighbor cells). (D) *stit* and *PI3K-I^DN^* co-expression resulted in smaller cells with autophagic puncta and low levels of p-4E-BP following starvation, indicating PI3K-I signaling lies downstream of Stit. (E) *Akt-IR*, (G) *RagA^DN^*, and (I) *TOR^TED^*-expressing cells are smaller, readily form autophagic puncta, and had lower or similar p-4E-BP levels to neighbor cells. (F) *Akt-IR*, (H) *RagA^DN^*, or (J) *TOR^TED^* co-expression in cells expressing *stit* inhibited the increase in cell size, resistance to starvation-induced autophagy (Ch::Atg8a puncta), or maintenance of p-4E-BP levels, indicating that these members of the PI3K-I/TORC1 signaling cassette are required for Stit-dependent starvation resistance. Scale bars, 25 µm. (K) Plot of the ratios of p-4E-BP labeling intensities in cells expressing the transgene to wild-type neighbor cells. The number of overexpressing cells/wild-type nearest neighbor cells counted (*n*) is given. * indicates Student's *t* test *p* values <0.005 between transgene-expressing and wild-type neighbor cells. Co-expression of *RagA^DN^* together with *stit* blocked *stit*-supported growth under starvation. This effect was so strong on p-4E-BP levels that it reduced levels far beyond the starved wild-type cell levels, giving significant differences. Inset *p* values compare differences in preservation of p-4E-BP signal between *stit*-expressing cells/wild-type neighbor cells and *stit* and transgene co-expressing cells/wild-type cells. Error bars indicate standard deviation.

To assay TORC1 activity, we examined the phosphorylation of the translational repressor 4E-BP by TORC1, upon *stit* overexpression. We generated clones of cells in the fat body of fed and starved larvae overexpressing *stit*, *RagA^DN^*, or *PI3K*-*CaaX* together with *GFP* and labeled the tissues with an antibody against p-4E-BP (Figures 4 and S4A) [22]. *RagA^DN^* expression, known to lower TORC1 activity, caused a reduction in p-4E-BP labeling in clones of fed larvae compared to neighboring cells (Figures 4K and S4A) [3]. Conversely, cells expressing *PI3K*-*CaaX* showed a stronger p-4E-BP signal than surrounding cells upon starvation (Figure 4A,K). As such, anti-p-4E-BP reflects the nutritional status and TORC1 activity levels of the cell.

Clonal Stit overexpression in fed larvae did not greatly affect p-4E-BP signal intensity (Figure 4K) but, like *PI3K*-*CaaX*, caused a robust increase of the signal in *stit*-expressing cells compared to wild-type neighbors following starvation (Figure 4A, B, and K). This indicates that *stit*, like InR, activation is sufficient to activate TORC1-mediated p-4E-BP phosphorylation and to protect cells against the starvation-induced drop in TORC1 activity. Next, we investigated whether dS6K, another well-characterized TORC1 effector, can be phosphorylated by Stit overexpression upon starvation. We analyzed fat body cell clones overexpressing *RagA^DN^* or *stit* with a p-dS6K antibody. *RagA^DN^* expression strongly reduced the cytoplasmic punctate p-dS6K staining observed in neighboring cells lacking *RagA^DN^* expression (Figure S4B and G). To assess the specificity of the cytoplasmic p-dS6K labeling, we overexpressed *dS6K* in fat body cell clones and subjected the larvae to a 5-h starvation period. We found that the p-dS6K signal was selectively enhanced in cells overexpressing *dS6K* compared to surrounding cells with endogenous dS6K levels (Figure S4C and G). This indicated that the p-dS6K antibody can faithfully recognize the cytoplasmic punctate accumulations of p-dS6K. To investigate the nature of these puncta, we labeled larvae expressing the late endosomal/lysosomal marker, GFP-Lamp1, with anti-p-dS6K. During the P.A. wave that clears the larval tissues at pupation GFP-Lamp1 puncta become enlarged and decorated by p-dS6K. This p-dS6K localization supports the observation that a major site of TORC1 activity is on late endosomal/lysosomal structures (Figure S4E) [5]. Clonal overexpression of *stit* in the fat body (Figure S4D and G) also resulted in the increase of p-dS6K puncta in starved larvae. This analysis indicates that Stit can induce the accumulation p-dS6K and p-4E-BP, two well-characterized TORC1 targets upon starvation.

We then monitored p-4E-BP accumulation, cell growth, and the autophagy reporter in clones of fat body cells expressing *stit* to ask if Stit acts through the conventional PI3K-I-TORC1 pathway to support growth and suppress starvation-induced autophagy. As previously established, clonal expression of *PI3K^DN^*, *Akt-IR*, and *RagA^DN^* in fat body cells all led to reduction of cell growth and p4E-BP levels and entry into autophagy (Figure 4C, E, and G) [3],[19]. Also, expression of *Tor^TED^* led to a strong cell size reduction, rendered cells equal to neighbors in respect to p4E-BP levels, while it induced a high Ch::Atg8a accumulation (Figure 4I). Co-expression of either *PI3K^DN^*, *Akt-IR*, *RagA^DN^*, and *Tor^TED^* together with *stit* reversed the effect of Stit in suppressing autophagy as well as its effect in sustaining growth and p-4E-BP levels upon starvation (Figure 4B, D, F, H, J, and K).

Collectively, the analysis of *stit* overexpression in endoreplicating tissues indicates that Stit can signal through the conventional PI3K-I/TORC1 pathway to sustain TORC1 signaling levels and block autophagy upon starvation.

### Stit Controls TORC1 Activity During Wing Development

**Figure 5:**
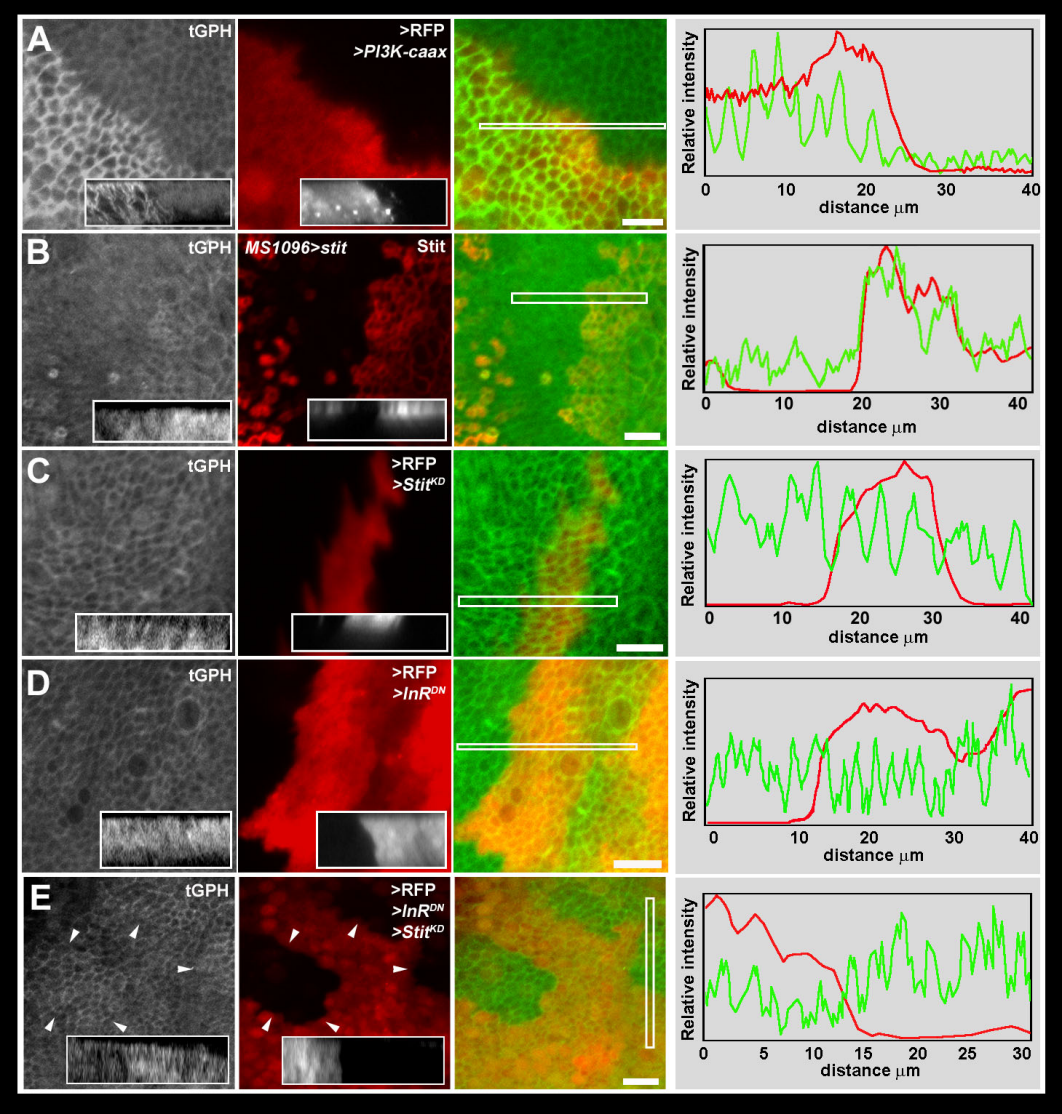
Stit and the insulin receptor cooperate to activate the PI3K-I pathway. (A) Clones of cells (marked with RFP) expressing *PI3K-CaaX* in the larval wing discs led to an increased recruitment of the tubulin::GFP-PH probe (tGPH) to the plasma membrane detectable following 24-h starvation. Inset X–Z section (3–4 µm) and line intensity graph is through/over the indicated region in merged panels. RFP (red) and GFP (green) intensities are represented as line intensity graphs. Peaks represent membrane-localized GFP, while troughs correspond to cytoplasmic signal. GFP-PH membrane-associated intensity in the PI3K-CaaX-expressing region is higher than the nonexpressing region. (B) MS1096 expression of *stit* (red) causes increased GFP-PH membrane recruitment in the expressing cells. (C) Clones of cells expressing *Stit^KD^* or (D) *InR^DN^* alone did not decrease membrane GFP-PH localization. (E) Clones expressing both *Stit^KD^* and *InR^DN^* had lower levels of membrane-localized GFP-PH than wild-type neighbors. Arrowheads denote clone boundaries. Insets show X–Z projections spanning the clones. All images are thin (8 µm) confocal projections. Scale bars, 10 µm.

To address whether Stit controls the growth of proliferating epithelial wing discs through the PI3K-I pathway, we first expressed *PI3K-CaaX* in clones using *hs-flp;Act>CD2>GAL4* (AFG4) and recorded the recruitment of the GFP-PH probe at the cell cortex. We detected a marked increase of the GFP-PH signal in the *PI3K-CaaX* cells, marked with RFP compared to adjacent nonexpressing cells under starvation conditions. This indicated that GFP-PH reliably reflects PI3K-I activation upon starvation in the wing (Figure 5A). Similarly to *PI3K-CaaX*, overexpression of *stit* by *MS1096*-GAL4 resulted in an increase of the GFP-PH signal along the membranes of the Stit overexpressing cells compared to their neighbors expressing endogenous levels (Figure 5B). This indicated that Stit could activate the PI3K-I pathway in the wing discs. However, neither the clonal inactivation of Stit nor interference with InR signaling by the expression of the dominant negative constructs was sufficient to induce a change in the intensity or the localization of the PI3K-I activity reporter (Figure 5C,D). By contrast, the concurrent expression of both *Stit^KD^* and *InR^DN^* using the same driver lead to a marked decrease in the GFP-PH intensity in the cells expressing both constructs compared to their neighbors (Figure 5E). This was most evident at the interface of cells expressing both *Stit^KD^* and *InR^DN^*. This suggests that Stit and InR co-operate to activate PI3K-I in the wing.

**Figure 6:**
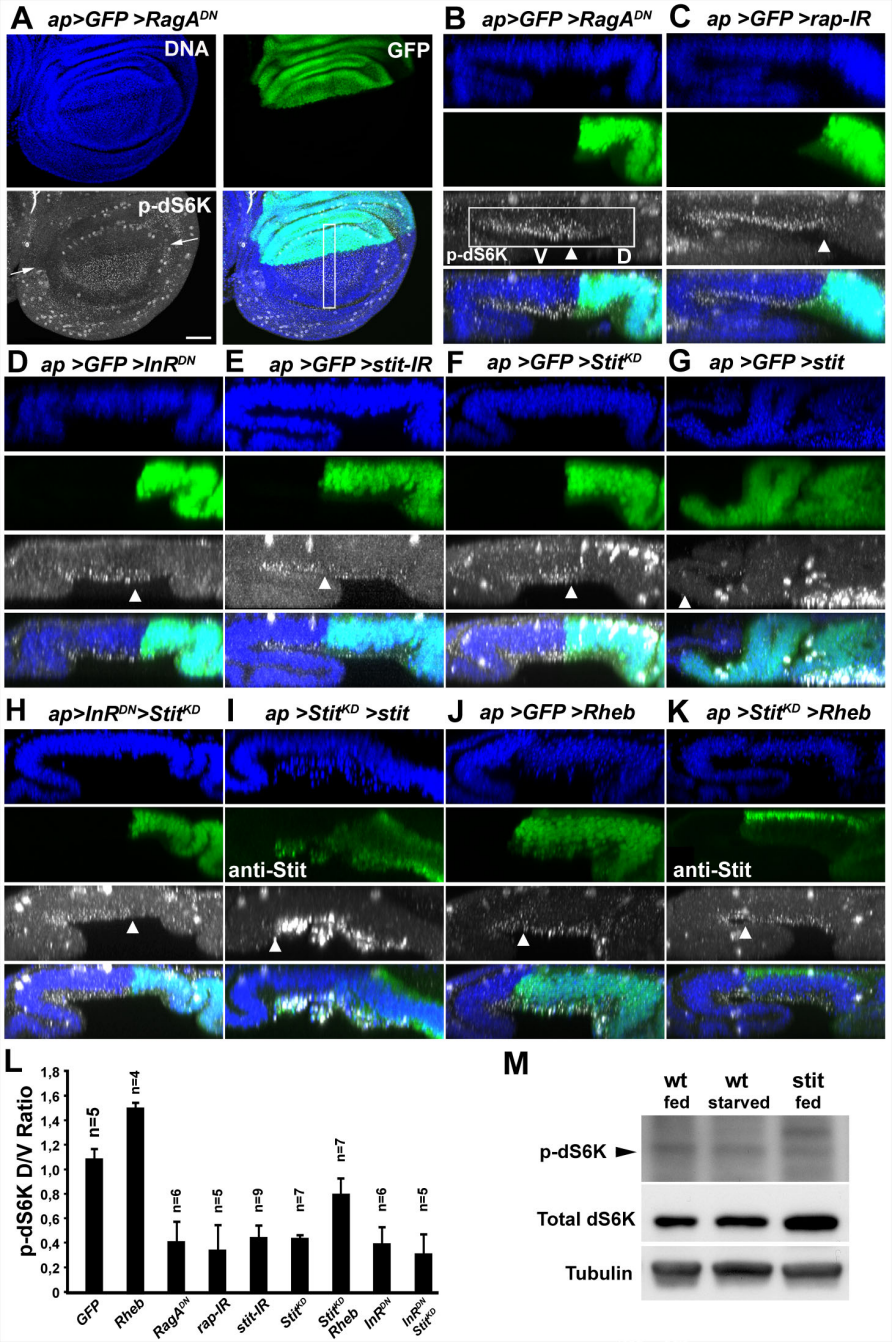
Stit and InR cooperatively govern TORC1 activity. Anti-p-dS6K labeling of third instar larval wing discs revealed discrete puncta lying basally within cells. See Figure S5 for control experiments addressing labeling specificity. Transgenes were expressed by *ap-GAL4* and expressing cells were identified by GFP (A–H and J) or by Stit detection (I, K). Arrows or arrowheads mark the D/V compartment boundary. D/V ratios of p-dS6K intensities are quantified in (L). (A) Expression of *RagA^DN^* led to a reduction in basal p-dS6K puncta in the dorsal compartment. X–Z sections of the boxed area are shown in (B). (B–F) X–Z sections of discs expressing *RagA^DN^*, *raptor-IR*, *InR^DN^*, *stit-IR*, or *Stit^KD^* in the dorsal compartment. The basal p-dS6K signal of interest is boxed in (B) and the dorsal (D) and ventral (V) compartments indicated. Dividing cells at the top of the panel also label strongly (see Figure S6). The dorsal compartment lies to the right in all X–Z sections and arrowheads mark the compartment borders. (G) Expression of *stit* caused overgrowth of the dorsal region of the disc accompanied by a change in disc morphology, preventing analysis of basal p-dS6K levels. (H) Co-expression of *Stit^KD^* and *InR^DN^* reduced the p-dS6K signal, to a similar extent as either inactivation alone; see (L) for quantification. (I) Co-expression of *stit* with *Stit^KD^* leads to a reduction of the *stit* overgrowth phenotype but gave excessively high p-dS6K levels precluding quantification. (J) *Rheb* expression increased p-dS6K levels within the dorsal region. (K) Co-expression of *Rheb* with *Stit^KD^* reverted the decrease in p-dS6K levels resulting from Stit inactivation (see L). Scale bar, 50 µm. (L) The intensity of basal p-dS6K within both dorsal and ventral compartments was measured and the D/V ratio for each genotype calculated. Student's *t* test showed *p*<0,001 when wild-type D/V ratios were compared with all genotypes except when compared to *ap>Stit^KD^>Rheb* (*p*<0.01). *ap>Stit^KD^>Rheb* was significantly different from *ap>Stit^KD^* (*p* = 0.0015). (M) Immunoblots of total lysates prepared from third instar larval wing discs of wild-type, wild-type starved, and *stit* mutant larvae. The ratio of p-dS6k/dS6K was reproducibly lower (40% average reduction (45% in M), 15% standard error, 5 independent experiments, 7 samples) in *stit* mutant discs compared to wild-type fed animals. Arrowhead indicates the band recognized by the anti p-dS6K antibody.

We further investigated the interplay of Stit and InR during wing growth by monitoring TORC1-dependent dS6K phosphorylation. We first asked whether *in situ* staining with the p-dS6K antibody provides a reliable readout for the detection of dS6K activation in the wing discs. We expressed dS6K in the dorsal compartment of the wing discs and stained for p-dS6K. We detected an increase of the p-dS6K signal selectively in the basal side of the epithelial cells expressing dS6K (Figure S5A). Conversely, dS6K inactivation by expression *dS6K-IR* in the posterior compartment of wing discs resulted in the reduction of the pdS6K basal signal in the posterior cells (Figure S5B). This prompted us to use the p-dS6K antibody for the *in situ* analysis of dS6K activation upon localized inactivation of Stit and members of the InR pathway in the wing discs. We expressed *RagA^DN^*, *raptor*-*IR* (an RNAi construct directed against the TORC1 component *raptor*), *InR^DN^*, *stit*-*IR*, and *Stit^KD^* together with *GFP* in the dorsal wing compartment and assessed their effects on dS6K phosphorylation. As expected, *InR^DN^*, *RagA^DN^*, or *raptor-IR* lead to a marked reduction in the intensity of p-dS6K labeling compared to the ventral compartment (Figure 6A, B, C, D, and L). Conversely, *Rheb* expression resulted in an increase in the intensity of p-dS6K labeling (Figure 6J,L). This indicates that the detected dS6K phosphorylation was responsive to the level of TORC1 activity in the compartment. The expression of *Stit^KD^* or *stit*-*IR* with the same driver resulted in a reduction of p-dS6K labeling to similar levels as the inactivation of InR pathway components (Figure 6D, E, F, and L). In accord with the *in situ* analysis of p-dS6K, we detected a modest but reproducible reduction of p-dS6K levels in Western blots of wing disc protein extracts deriving from *stit* mutant larvae compared to wild-type controls (Figure 6M). The reduction in dS6K phosphorylation upon Stit or InR pathway component inactivation is in close agreement with the adult wing morphology defects caused by the same constructs. This indicates that Stit and InR in parallel control epithelial tissue growth by inducing dS6K phosphorylation.

To address possible intersection points of InR and Stit signaling, we overexpressed Rheb and inactivated Stit in the same cells using *ap-GAL4*. *UAS-Rheb* expression ameliorated the decrease in p-dS6K labeling caused by Stit inactivation (Figure 6F, K, and L). The restoration of p-dS6K staining intensity to 80% of wild-type together with the strong suppression of the adult wing-bending phenotype caused by Rheb expression in Stit-deficient cells indicate that Stit acts through Rheb to activate TORC1 during wing development (Figures 6K,L and 2A). We concluded that Stit and InR collectively activate PI3K-I and regulate TORC1 levels in the wing imaginal disc to control tissue growth.

### Stit and InR Cooperatively Prevent the Catabolic Switch to Autophagy

**Figure 7:**
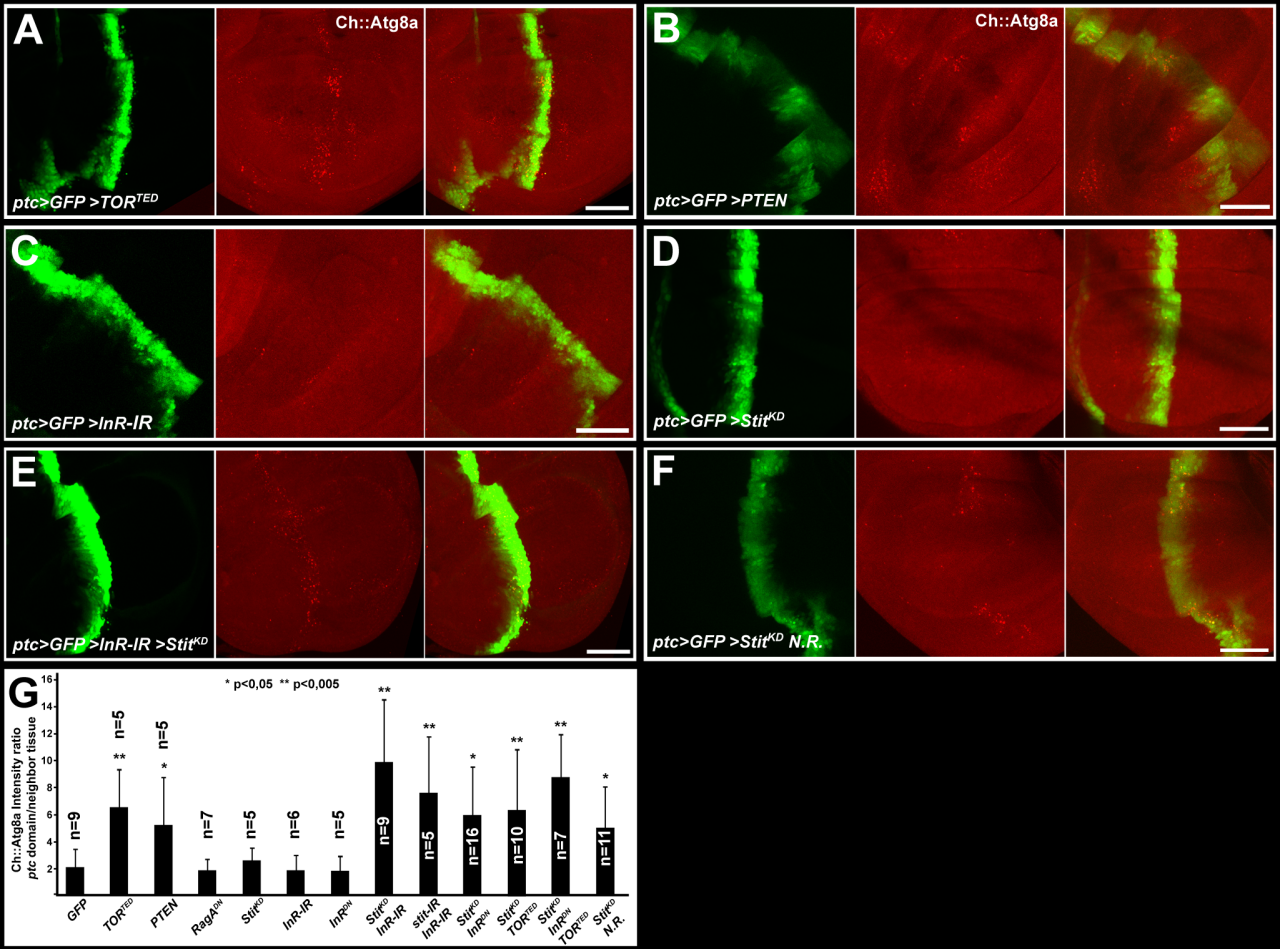
Stit and InR are interchangeably sufficient to block autophagy. *ptc*-*GAL4* was used to drive transgenes and GFP in the wing discs of third-instar larvae expressing a Cherry-tagged Atg8a autophagy reporter under the control of its own promoter (*Ch::Atg8a*). GFP expression did not affect the formation of Ch::Atg8a puncta (see Figures S6A and 7G). (A) Expression of a dominant negative form of TOR (*TOR^TED^*) led to a robust induction of autophagic puncta compared to wild-type neighboring tissue. Quantified in (G). (B) Expression of *PTEN* induced the formation of autophagic puncta, indicating PI3K-I signaling normally holds the autophagic machinery dormant. Expression of either (C) *InR-IR* or (D) *Stit^KD^* in the *ptc* domain did not trigger an increase in punctate Ch::Atg8a accumulation. (E) Co-expression of *InR-IR* together with *Stit^KD^* led to a robust increase in the formation of autophagic Atg8a puncta. Comparable levels of autophagic puncta were observed upon co-reduction of InR/Stit signaling via double RNAi or double dominant negative approaches (see G and Figure S6). (F) Rearing *Stit^KD^*-expressing animals on nutrient-restricted (N.R.) low-energy food led to a notable induction of autophagic puncta, quantified in (G). Scale bar, 50 µm. (G) Graph displaying the ratios obtained from comparison of Ch::Atg8a intensities within the *ptc*-GFP stripe with the flanking tissue. Student's *t* test scores for significant differences to control are indicated.

InR and Stit control TORC1 activity during wing growth while Stit can block autophagy in endoreplicating larval tissues in response to starvation. To directly monitor starvation-induced autophagy in the wing, we used the *Ch::Atg8a* reporter under the control of its endogenous promoter. We first expressed either *GFP* alone, or *TOR^TED^*, or *RagA^DN^* or *InR^DN^* or *PTEN* together with *GFP* using *ptc*-*GAL4*. While GFP alone had no effect on the induction of autophagy (Figures S6A and 7G), both *TOR^TED^* and *PTEN* caused a marked increase in the number of Ch::Atg8a puncta forming within the GFP marked expression domain (Figure 7A, B, and G), indicating that the PI3K-I/TORC1 axis functions to suppress autophagy in the wing. Surprisingly, *RagA^DN^* and *InR^DN^* or *InR-IR* showed little or no accumulation of Ch::Atg8a puncta in the *ptc* expression domain (Figures 7C,G and S6B) despite each causing a substantial decrease in the levels of TOR target p-dS6K (Figure 6 and unpublished data). This argues that p-dS6K phosphorylation and the activation of autophagy markers respond to different levels of InR and TORC1 activity in the wing. This contrasts the analysis of TORC1 activation in the fat body, where InR signaling is the dominant receptor activating PI3K-I. In this tissue, InR mutant cells or cells expressing *InR-IR* or *InR^DN^* both restrict growth and activate the catabolic process of autophagy (Figure S3E, F) [19]. Like the InR, Stit inactivation by either *Stit^KD^* or *stit*-*IR* using *ptc*-*GAL4* (Figure 7D, G and unpublished data) did not increase Ch::Atg8a puncta formation. This analysis indicates that although inactivation of TORC1 alone leads to autophagy, inactivation of either of its upstream receptors is not sufficient to induce the catabolic response in epithelial proliferating cells.

**Figure 8:**
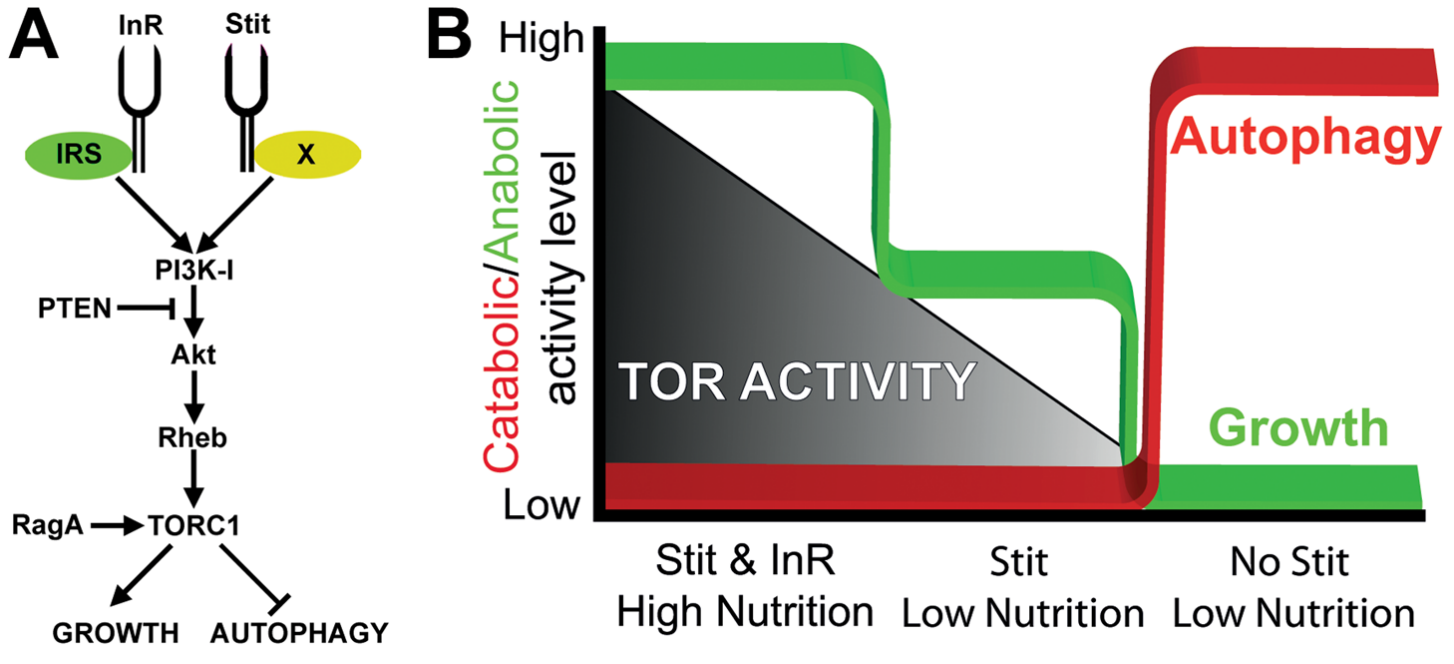
A model for the different modes of autophagy regulation in tissues that express *stit*. (A) A model depicting Stit and its intersection with the InR/PI3K-I/TORC1 pathway. As Stit is required and sufficient for PI3K-I activation, we place it in parallel to the InR. While InR is known to signal through Chico (IRS) and Lnk, the RTK adaptor that couples Stit to PI3K-I signaling is unknown (X). (B) The presence of *InR* or *stit* is sufficient to block autophagy in the wing, while both are required for optimal wing growth. When reared on low-energy food, Stit is required for repressing autophagy. These findings predict a two-tiered model for the switch between anabolism to catabolism in epithelial tissues.

We hypothesized that InR and Stit signaling may cooperatively drive TORC1 activation, and hence only a reduction of both pathways would mimic the effect of *TOR^TED^* on autophagy. We introduced either *Stit^KD^ InR^DN^* or *Stit-IR InR-IR* or *Stit^KD^ InR-IR* into the *ptc*-GAL4 *Ch::Atg8a* background to examine the effect of inactivating both receptors on autophagy (Figures 7E, G and S6). Each manipulation resulted in a striking increase in the levels of Ch::Atg8a puncta comparable to the one caused by *TOR^TED^* expression. Finally we challenged *ptc*>*Stit^KD^* animals with prolonged nutrient restriction on low-energy food. This regime delays development by 2–3 d but is sufficient to support larval development to adulthood. Remarkably, constant nutrient restriction gave an increase in autophagy specifically within the *ptc* domain upon reduction of Stit signaling (Figure 7F, G). We conclude that Stit and InR are interchangeably required to sustain TORC1 activity and to prevent a catabolic switch in wing discs (Figure 8). Thereby, they endow proliferating imaginal epithelial tissues with a two-tiered control of growth and autophagy.

## Discussion

Animals modulate organ-specific growth according to their developmental stage and homeostatic needs. This is particularly evident during nutrient starvation, when organisms respond by relocating stored energy resources and by recycling of cellular material. The starvation response is, however, not equal in every organ in regards to growth (anabolism) and shrinkage (catabolism) and the molecular mechanisms involved in these different responses are poorly understood. Several findings suggest that variations of insulin-signalling-mediated growth are at play in different organs. In adult *Drosophila*, the size of the gut is dynamically regulated depending on food availability and InR signaling [29]. Another recent study revealed that growth can be controlled by an alternative RTK when Insulin signaling is reduced in the developing larval *Drosophila* brain [22]. When larvae were cultivated under nutrient-restricted conditions and InR signaling was low, neuroblasts (NBs) were still able to proliferate. This “brain sparing” is dependent on the ALK receptor in neuronal lineages and its ligand Jelly Belly, which is expressed in glia [30]. Importantly, under normal feeding conditions, ALK is essential for NB development, showing that the ALK signaling pathway is not merely a general backup for the InR upon low nutrition; it rather promotes the growth of specific NB lineages at low InR signaling levels. In this respect, Stit, like ALK, supports growth under variable nutrient conditions, promoting proliferation in epithelial tissues upon low InR signaling but is also necessary for optimal epithelial growth under normal conditions. Curiously, the signaling pathways supporting growth and proliferation downstream of ALK and Stit appear to be different. TSC1/2, Rheb, and TORC1 were dispensable for ALK function in growth and proliferation, while the direct TORC1 downstream targets S6K and 4E-BP were required. Stit, in contrast to ALK, utilizes the classical PI3K-I/TORC1 pathway to drive growth. First, Stit is sufficient to drive PI3K-I activation and suppress autophagy in starved fat body cells. Second, the suppression of autophagy is rapamycin-sensitive and hence TORC1 dependent. Third, Stit and InR cooperate to control PI3K-I activity and autophagy suppression in the wing, and finally, *PI3K-I* and *Rheb* overexpression can rescue Stit inactivation phenotypes. As ALK does not signal through TORC1, it is hence unlikely to regulate autophagy. Thus, apart from the insulin receptor, Stit provides the first example of an RTK that negatively regulates autophagy.

Simultaneous reduction of the signaling activity of both Insulin and Stit receptors, or prolonged starvation together with reduced Stit activity, leads to the induction of autophagy in the wing. We propose that the simultaneous inactivation of Stit and InR reduces PI3K-I activity and TORC1 signaling to a critically low level, beyond the limit of our p-dS6K detection range. This reveals a mechanism where Stit or InR signaling prevent TORC1 activity from dropping below a threshold where it can no longer suppress autophagy. Thus, while anabolic growth can vary in response to RTK signaling, autophagy and catabolism in proliferating epithelia are strictly inhibited by signaling from either Stit or InR. The cooperative functions of Stit and InR provide a novel failsafe mechanism, allowing TORC1 activity to variably modulate growth under fluctuating nutrient conditions without incurring a transition to catabolism (Figure 8). As Stit is selectively expressed in imaginal discs giving rise to adult epithelial organs, it may function to safeguard the growth of these tissues during conditions of low nutrient availability, at the expense of nonexpressing tissues.

The product of the mammalian Ret oncogene and Stit share several distinctive features. Their amino acid sequences are 42% identical and 64% homologous within the kinase domain. Both Stit and Ret are composed of an extracellular region with a Cadherin domain, a transmembrane stretch, and an intracellular tyrosine kinase domain. Apart from Stit the fly genome encodes a second Ret paralog (dRet) predominantly expressed in neurons. Although the signals that activate the *Drosophila* Ret-like proteins remain unknown, Stit is activated upon epidermal wounding to initiate re-epithelialization and barrier repair. Mammalian Ret is activated by GDNF to instruct epithelial morphogenesis in the uteric duct of the kidney [31] and Ret-activating mutations have been implicated in a variety of human cancers including epithelial cancers (breast and lung) and multiple endocrine neoplasia (*men2*) [32]–[35]. More recently, overexpression of an activated form of *Drosophila* Ret that mimics the mutation that leads to *men2* has been used to identify potential Ret signal transducers and drugs that interfere with its aberrant activation [36]. Our analysis reveals the physiological role of Stitcher in epithelial tissue growth and proliferation and strengthens the notion that Stit and Ret share the same functions in controlling PI3K-I and TORC1 activity in epithelial tissues. The newly identified function of Stit in sparing proliferating epithelial organs from starvation-induced autophagy raises the question of whether Ret activation may suppress autophagy as well. Since oncogenic Ret mutations promote cancer growth in part by activation of the TORC1 pathway [36],[37], our findings suggest that aberrant Ret signaling may suppress autophagy during cancerous growth, potentially providing an advantageous mechanism or driving force for the growth of Ret-expressing tumors.

## Experimental Procedures

### Fly Cultivation and Stocks

Flies were cultivated at 25°C on our standard lab fly medium consisting of, per liter, 32.7 g dried potato powder, 60 g sucrose, 27.3 g dry yeast, 7.3 g agar, 4.55 ml propionic acid, and 2 g nipagin, giving a final concentration of 15.3 g/l protein and 6 g/l sugar. Low-energy food consisted of 35 g dried potato powder, 10 g glucose, and 8 g agar per liter. For starvation experiments, larvae were transferred to PBS Agar (1%) for defined periods. Rapamycin (Santa Cruz Biotech, sc-3504A resuspended in Methanol) was diluted to 100 uM in 20% Sucrose PBS and mixed to a paste 1∶1 with dry yeast, which was added to PBS agar vials to which larvae were placed for defined periods.

The fly stocks *w^1118^*, *ap-GAL4/CyO*, *MS1096-GAL4* [38], *en-GAL4*, *da-GAL4*, *UAS-InR^DN^* (K1409A), *UAS-InR-CA*(R418P), *UAS-InR-IR*, *UAS-Rheb*, *UAS-PTEN*, *UAS-PTEN-IR*, *raptor-IR*, *UAS-Akt*, *UAS-PI3K-CaaX*, *UAS-PI3K-I*, *UAS-dS6K alleles* (wild type and constitutive active, S6K^TE^, S6K^STDE^, or S6K^STDETE^, which are intrinsically active due multiple serine/threonine to acidic amino acid substitutions [39]), *UAS-TOR^TED^*, *UAS-CycB*, *UAS-CycE.L*, *CycE^AR95^*, *dap^4^*, *UAS-FLP*, *FRT82B Ubi-GFP*, *UAS-GFP*, *hs-flp; FRT82B*, *yw hs-flp;Dr/Tm3,Sb(1)*, y,w, *hs-flp; Act>CD2>GAL4; UAS-GFP*, and *UAS-p35* were from Bloomington, while *UAS-stit-IR* (1091 and 8401) and *UAS-dS6K-IR* (18126) were from VDRC. *UAS-Stit^KD^*, *UAS-stit*, *stit-α-gfp*, *stit^ex266^*, and *stit^EXEL9056^* were described previously [24]. The pWIZ RNAi vector was used to generate the UAS-*stit-IR^D158^* that targets the third exon of *stit*. Other stocks included *hs-flp,UAS-RFP* (kind gift from D. Hipfner), *UAS-RagA* ^T16N^, designated *RagA^DN^*, *act>CD2>GAL4/CyO;tGFP-PH* (gift from Stephen M. Cohen) [40], *pmCh::Atg8a/CyO* [41], *yw FLPf36a; FRT82B UbqGFP 83f+ 87D M(3)95A/Tm6b, Tb(1)* (gift from Dr. Fernando Roch), and *hsp70-Flp; UAS-Dicer; r4-mCh::Atg8a*, *act>CD2>GAL4*, and *UAS-GFPnls* (generously provided by Thomas Neufeld). The last stock has a leaky heat shock promoter requiring no heat shock, otherwise 20 or 75 min heat shock in a 37°C water bath was applied for fat body or wing clones, respectively. *hs-flp*; *act >CD2>GAL4*–generated clones are denoted as AFG4.

### Immmunohistochemistry and Microscopy

Larval fat body and/or discs were dissected from larvae, fixed in 4% formaldehyde/PBS (either Sigma F1635 or polysciences #18814 ultrapure) for 20 min, and labeled immediately afterward following standard protocols (PBSBT containing 0.5% BSA). Primary anti-sera included rabbit anti-GFP (Invitrogen, 1/300), rabbit anti-p-dS6K and p-4E-BP (Cell signaling #9209 and #2855, each diluted 1/100), rabbit anti-PH3 (sigma, 1/200), rabbit anti-CycB (D. Glover, 1/750), rat anti-dGem (H. Richardson, 1/300), mouse anti-Fas3 (1/100), and rat anti-DE-Cad (1/30) from DSHB and guinea-pig anti-Stit (1/5,000) [24]. Secondary antibodies were from Jackson Immunolab and Molecular probes. TUNEL and EdU assay kits were from Roche and performed as detailed therein. Samples were mounted in Vectashield H-1000 (vectorlabs) for imaging on either Zeiss LSM510, 710, or 780 confocal microscopes or Zeiss Axioplan2. Western blotting was performed using HRP-conjugated antibodies (Jackson Immunolabs). Rabbit p-dS6K (as above) was used at 1/1,000, mouse anti-tubulin (sigma, T5168) was used at 1/100,000, while rabbit anti-dS6K (kind gift of T. Neufeld) was used at 1/1,000. Twenty larval wing discs per well were run on 10% mini-PROTEAN TGX gels (Biorad). Wild-type animals were starved for 48 h on PBS agar. Densitometric analysis was performed using ImageJ and Image Studio Lite Western Blot Analysis Software (LI-COR).

### Image Analysis

Image analysis was performed using ImageJ. For DE-Cad pupal wing labeling and PH3 mitotic cell counting in the wing, a cell counting grid was applied to a projection of the confocal stack to aid manual counting. In the case of pupal wing cell quantification, projections of the dorsal or ventral apical cell surfaces immediately overlaying each other were analyzed. GAL4-driven expression of GFP or RFP allowed distinction of cells of interest in all cases. For intensity measurements (e.g., p-dS6K), thin (10 µm) projections of stacks were analyzed. In brief, the threshold function was applied to images and the dorsal and ventral regions (or in the fat body cells expressing GFP versus their immediate neighbors) were manually highlighted and the intensities measured using the analyzed particles function. Care was taken to ensure that control cells or regions of tissues from different samples and genotypes gave similar background values to which test cells or regions were compared. From this the intensity relative to area was determined for both background and test regions. For AFG4 clones, the number of GFP-positive cells within a clone was manually counted from confocal stacks spanning the entire disc. CDT was calculated using the formula (log2/log*n*)*h*, where *n* = median number of cells per clone and *h* = age of the clone [25]. The adult wing hair measurements were aided by the image J. In Figure S1C, the posterior region is defined as the area from L4 to posterior wing margin and the anterior region is defined as the area from L3 to anterior wing margin. Error bars in all figures represent standard deviation from the mean. All *p* values were generated by student's *t* test.

## Supporting Information

Figure S1 Stit is expressed in the wing and required for its optimal growth. (A) *ap-GAL4* was used to induce FRT *stit* mutant clones (marked by the absence of GFP) in third-instar larval wing discs. Stit protein was not detectably decreased in clones (anti-Stit labeling in red). Scale bar, 50 µm. (B) Pupal wing discs from the same genotype as in (A). Thirty hours after pupal formation (APF), Stit protein was undetectable in GFP negative *stit* clones. Scale bar, 50 µm. (C) Quantification of relative cell numbers estimated by comparing the growth (counting wing hairs) of *f*-marked wild-type and *f*-marked *stit* mutant clones opposing minute mutant cells. Wild-type clones near entirely outgrew minute cells and were assigned a relative cell number of 1 to which *stit* versus minute was compared. The number of wings counted (*n*) is indicated. (D) DE-Cadherin labeling of pupal wings expressing *Stit^KD^* in the dorsal compartment. The number and height (X-Z sections below) of dorsal cells was reduced compared to the ventral cells (see also Figure 1). *ap>GFP* wings served as controls. Scale bar, 10 µm. (E) Labelling control or *ap>Stit^KD^* third-instar larval wing discs with EdU (left) or dGeminin, (right) revealed an overall decrease of labeling both in dorsal and ventral compartments, quantified in the graph. The number of wing discs included in the analysis (*n*) is indicated. Student's *t* test values inset. (F) The expression of *Stit^KD^* in the dorsal compartment led to an accumulation of Cyclin B in the ventral compartment, indicative of cell cycle arrest or delay. This was not observed in the controls (*ap>GFP*), quantified in the graph. The number of wing discs analyzed is indicated. (G) Control or *ap>Stit^KD^* third-instar larval discs were labeled with TUNEL to detect apoptosis. No notable difference in labeling was detectable between the two genotypes (*p*>0.5). Arrowheads in (E–G) mark the D-V boundary. The number of wing discs examined is indicated. Scale bar, 50 µm in (E–G).
(TIF)

Figure S2 Stit overexpression leads to tissue overgrowth. (A) Low-level expression of *stit* in the dorsal domain (18°C) led to mild and reproducible downwards bending of the adult wing. Expression at higher temperatures resulted in lethality. (B) *ap>stit* discs labeled for Stit, BrdU, and PH3. The signal for the proliferation markers BrdU and anti-PH3, is increased in the *stit*-expressing domain (outlined with a hatched line) compared to the rest of the disc.
(TIF)

Figure S3 InR but not Stit kinase activity supports growth and suppresses autophagy in the fat body. Inactivation of InR and/or Stit in clones of larval fat body cells (labelled by RFP). (A–D) Clonal disruption of InR (*InR^DN^*) or Stit (*Stit^KD^*), separately or together, had no detectable effect on PI3K-I activity as judged by the tGPH probe (green). (E, F) InR inactivations cause the punctate accumulation of the Ch::Atg8a autophagy reporter in the expressing cells. DNA staining reveals the reduced ploidy/cell size in *InR^DN^*- or *InR*-*IR*-expressing cells (GFP positive and hatched outline). (G) Fat body clones expressing *Stit^KD^* (GFP positive and hatched outline) did not induce changes in the accumulation of the autophagy marker in FB cells. (H) Effects of both *InR-IR* and *Stit^KD^* expression in clones were comparable to *InR*-*IR* alone. Scale bar, 50 µm.
(TIF)

Figure S4 The PI3K-I/TORC1 signaling cassette is required for Stit-dependent protection against starvation. (A) Clones of fat body cells expressing *RagA^DN^* (marked with GFP in green) showed reduced p-4E-BP levels under fed conditions. (B) Cells expressing *RagA^DN^* (GFP positive cells outlined with hatched line) showed lower p-dS6K labeling, while wild-type neighbor cells displayed a punctate cytoplasmic labeling pattern. The nuclear p-dS6K labeling was unaltered under these and other conditions (see C and D) and is therefore likely unspecific. Scale bar, 50 µm. (C) Cell clones overexpressing *dS6K* in larvae under starvation showed increased levels of cytoplasmic p-dS6K signal, while the nuclear labeling remained unaltered. Scale bar, 50 µm. (D) *stit* expression in cell clones showed increased cytoplasmic accumulation of p-dS6K under starvation conditions. (E) The cytoplasmic puncta of p-dS6K coincided with the late endosomal/lysosomal GFP-tagged protein Lamp1, coating the exterior of the lysosome, in fat body cells undergoing programmed autophagy (P.A.). Inset shows an enlargement of the highlighted area. Scale bar, 10 µm. (G) The intensity of p-dS6K in cells overexpressing *dS6K* (following 5-h starvation), *RagA^DN^*, *stit* (following 24-h starvation), and neighboring wild-type cells was quantified and the ratios were calculated and plotted. The number of overexpressing/neighbor cells (*n* = overexpressing cells/neighbor cells) quantified is indicated. *indicates significant *p* values (<0.005) between overexpressing cells and nearest neighbor cells. Error bars indicate standard deviation.
(TIF)

Figure S5 Stit is required for optimal TORC1 signaling in the wing. (A) *dS6K* overexpression in the dorsal compartment led to a selective basal accumulation of p-dS6K puncta in the dorsal cells of third-instar larval wing discs (right and X–Z section). Dashed line or arrowhead (X–Z section) marks the D/V compartment boundary. Larger clusters of p-dS6K located apically (left) correspond to mitotic cells, magnified in the far right lower panel. Magnified panel measures 25×25 µm. (B) Expression of *dS6K-IR* in the posterior wing compartment (labelled by GFP) via *en-GAL4* lead to a reduction of the basal p-S6K positive puncta, indicating its specificity. The labeling of apical mitotic cells appeared unchanged. Hatching and arrowheads (X–Z section) mark the position of the compartment boundaries in (B) and (C). The basal location of the p-dS6K signal is indicated with a directional arrowhead in X–Z sections. (C) Expression of *Stit^KD^* in the posterior compartment led to a reduction of the basal p-dS6K signal in the posterior compartment. Scale bar, 50 µm.
(TIF)

Figure S6 Stit and InR are required to suppress autophagy in the wing. (A) Expression of GFP in the *ptc* domain of third-instar wing discs did not cause a notable change in the accumulation of *Ch::Atg8a* expressed under the control of its own promoter. The regions selected for intensity quantification (GFP positive and neighbor region) are indicated. (B) Knock-down of InR (*InR^DN^*) in the *ptc* domain did not change *Ch::Atg8a* accumulation. (C) Co-expression of *InR-IR* and *stit-IR* induced an increase in punctate *Ch::Atg8a* accumulation. (D) Expression of *TOR^TED^* together with *Stit^KD^* did not lead to a further increase in autophagy compared to *TOR^TED^* alone (Figure 7A). See Figure 7G for quantification. Scale bar, 50 µm.
(TIF)

Table S1 Phenotype strength was graded from severe (++++) to nil (−) unless not determined (ND). Additional phenotypes such as lethality (L) or semilethality (S.L.) were also noted. The percentage flies showing the grade of phenotype is given, as is the number of flies displaying the phenotype when totaling less than 50. Otherwise, greater than 50 flies were examined. Second or third indicates the chromosome the transgene is inserted on when more than one transgene stock was tested. All dS6K transgenes were mutated with different point mutations to be constitutively active. * An extremely small crumpled wing phenotype, while U denotes a bent upward wing and D a bent downward wing.
(DOCX)

## Abbreviations

4E-BP: Eukaryotic translation initiation factor 4E-binding protein 1
Act: Actin promoter
AFG4: Actin flp-out Gal4
Akt: Ak strain transforming
ALK: Anaplastic Lymphoma Kinase
ap: apterous
Atg8a: Autophagy-related gene 8a
ATP: adenosine triphosphate
BrdU: 5-bromo-2′-deoxyuridine
CaaX: farnesyltransferase and geranylgeranyltransferase I target site
CD2: stop sequence of the CD2 gene
CDT: cell doubling time
Ch: Cherry-tagged
CycB: Cyclin B
CycE: Cyclin E
da: daughterless
Dap: Dacapo
DE-Cad: Drosophila E-Cadherin
dGem: Drosophila Geminin
dILPs: Drosophila Insulin-like peptides
DN: dominant negative
dS6K: Drosophila ortholog of S6K
D/V: dorsal/ventral
EdU: 5-ethynyl-2′-deoxyuridine
en: engrailed
f: forked
Fas3: Fasiclin 3
FLPase: Flippase
GDNF: Glial cell-derived neurotrophic factor
GFP: green florescent protein
GFP-PH: GFP tagged PH domain
GTR1: GTP-binding protein GTR1
HS: heat shock
IGF: insulin growth factor
InR: insulin receptor
IR: interfering RNA
IRS: insulin receptor substrate
KD: kinase dead
Lamp1: lysosomal-associated membrane protein 1
Lnk: adaptor protein *SH2*-B3
men2: multiple endocrine neoplasia
MS1096: Gal4 driver
Myc: Myc proto-oncogene protein
NBs: neuroblasts
N.R.: nutrient-restricted
p-4E-BP: phosphorylated 4E-BP
P.A.: programmed autophagy
p-dS6K: phosphorylated dS6K
PDK1: Phosphoinositide-dependent kinase-1
PH: pleckstrin homology
PH3: phosphohistone H3
PI3K-I: Phosphatidylinositol 3-kinase class I
PIP3: phosphatidylinositol 3
4
5 trisphosphate
PRAS40: proline-rich Akt/PKB substrate 40 kDa
ptc: patched
PTEN: Phosphatase and tensin homolog
RagA: RagA protein
Rap: Raptor
Ret: Rearranged during trasfection
RFP: Red florescent protein
Rheb: Ras homolog expressed in brain
RNAi: RNA interference
RTK: receptor tyrosine kinase
S6K: Ribosomal subunit S6 kinase
Stit: Stitcher
stit-IR: stit interfering RNA
tGPH: tubulin driven GFP tagged PH domain
TORC1: target of rapamycin complex 1
Tor^TED^: Target of rapamycin toxic effector domain
TSC1/TSC2: tuberous sclerosis complex 1/tuberous sclerosis complex 2
TUNEL: *Terminal* deoxynucleotidyl transferase dUTP nick *end* labeling
UAS: upstream activating sequence
