# Supplementary figures and images for: Two-Tiered Control of Epithelial Growth and Autophagy by the Insulin Receptor and the Ret-Like Receptor, Stitcher
Source: PLoS Biol. 2013 Jul 23;11(7):e1001612. doi: 10.1371/journal.pbio.1001612 (PMC3720245; doi:10.1371/journal.pbio.1001612)

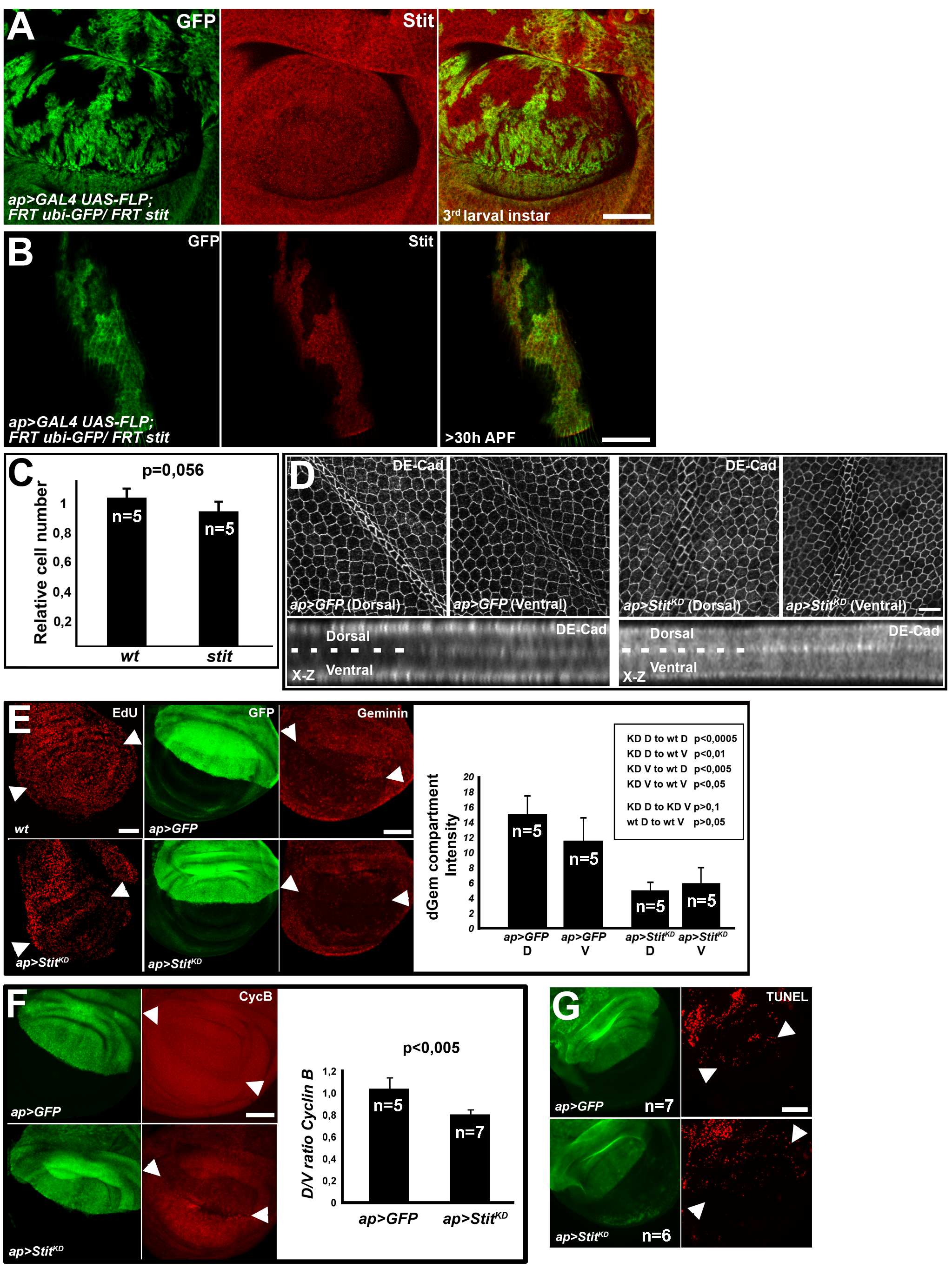

Supplement: Figure S1 — Stit is expressed in the wing and required for its optimal growth. (A) ap-GAL4 was used to induce FRT stit mutant clones (marked by the absence of GFP) in third-instar larval wing discs. Stit protein was not detectably decreased in clones (anti-Stit labeling in red). Scale bar, 50 µm. (B) Pupal wing discs from the same genotype as in (A). Thirty hours after pupal formation (APF), Stit protein was undetectable in GFP negative stit clones. Scale bar, 50 µm. (C) Quantification of relative cell numbers estimated by comparing the growth (counting wing hairs) of f-marked wild-type and f-marked stit mutant clones opposing minute mutant cells. Wild-type clones near entirely outgrew minute cells and were assigned a relative cell number of 1 to which stit versus minute was compared. The number of wings counted (n) is indicated. (D) DE-Cadherin labeling of pupal wings expressing StitKD in the dorsal compartment. The number and height (X-Z sections below) of dorsal cells was reduced compared to the ventral cells (see also Figure 1). ap>GFP wings served as controls. Scale bar, 10 µm. (E) Labelling control or ap>StitKD third-instar larval wing discs with EdU (left) or dGeminin, (right) revealed an overall decrease of labeling both in dorsal and ventral compartments, quantified in the graph. The number of wing discs included in the analysis (n) is indicated. Student's t test values inset. (F) The expression of StitKD in the dorsal compartment led to an accumulation of Cyclin B in the ventral compartment, indicative of cell cycle arrest or delay. This was not observed in the controls (ap>GFP), quantified in the graph. The number of wing discs analyzed is indicated. (G) Control or ap>StitKD third-instar larval discs were labeled with TUNEL to detect apoptosis. No notable difference in labeling was detectable between the two genotypes (p>0.5). Arrowheads in (E–G) mark the D-V boundary. The number of wing discs examined is indicated. Scale bar, 50 µm in (E–G). (TIF) [file pbio.1001612.s001.tif]

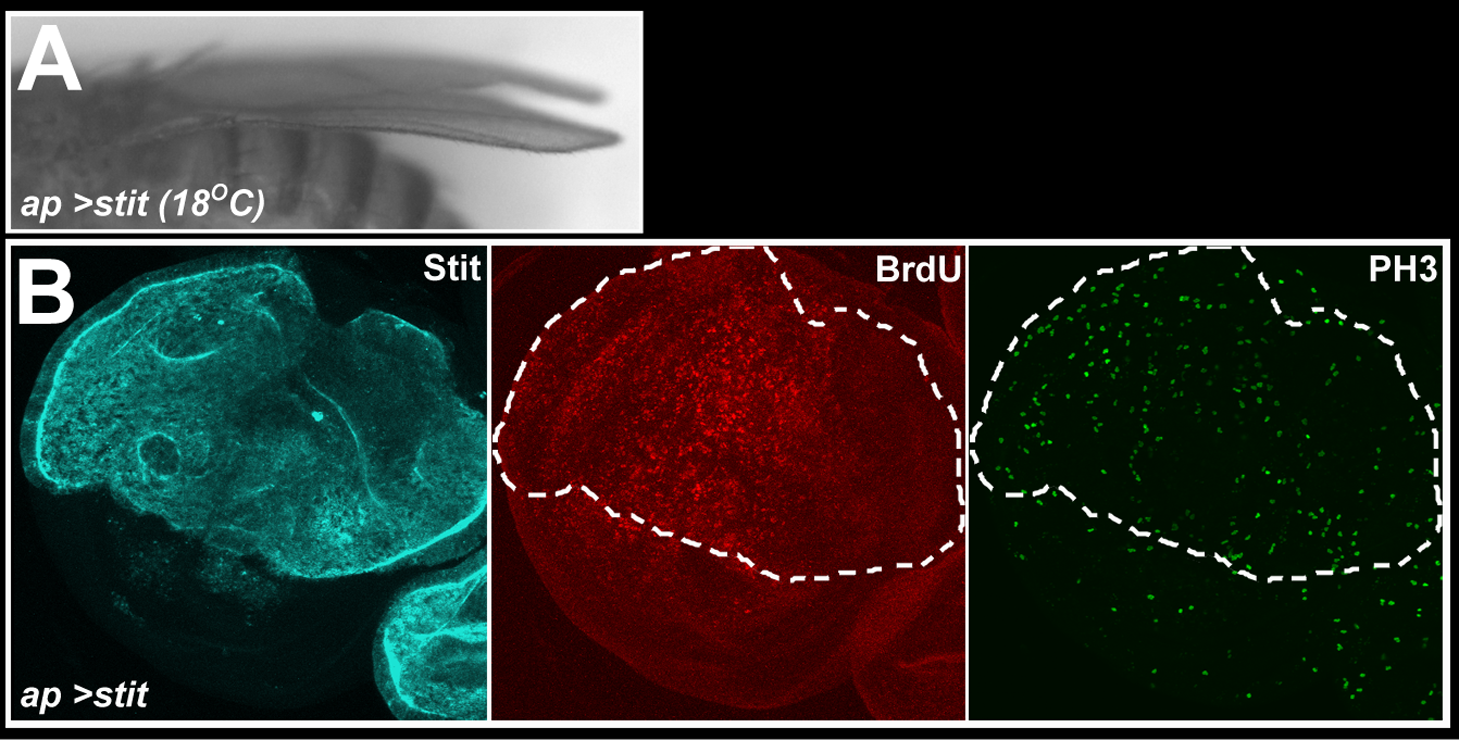

Supplement: Figure S2 — Stit overexpression leads to tissue overgrowth. (A) Low-level expression of stit in the dorsal domain (18°C) led to mild and reproducible downwards bending of the adult wing. Expression at higher temperatures resulted in lethality. (B) ap>stit discs labeled for Stit, BrdU, and PH3. The signal for the proliferation markers BrdU and anti-PH3, is increased in the stit-expressing domain (outlined with a hatched line) compared to the rest of the disc. (TIF) [file pbio.1001612.s002.tif]

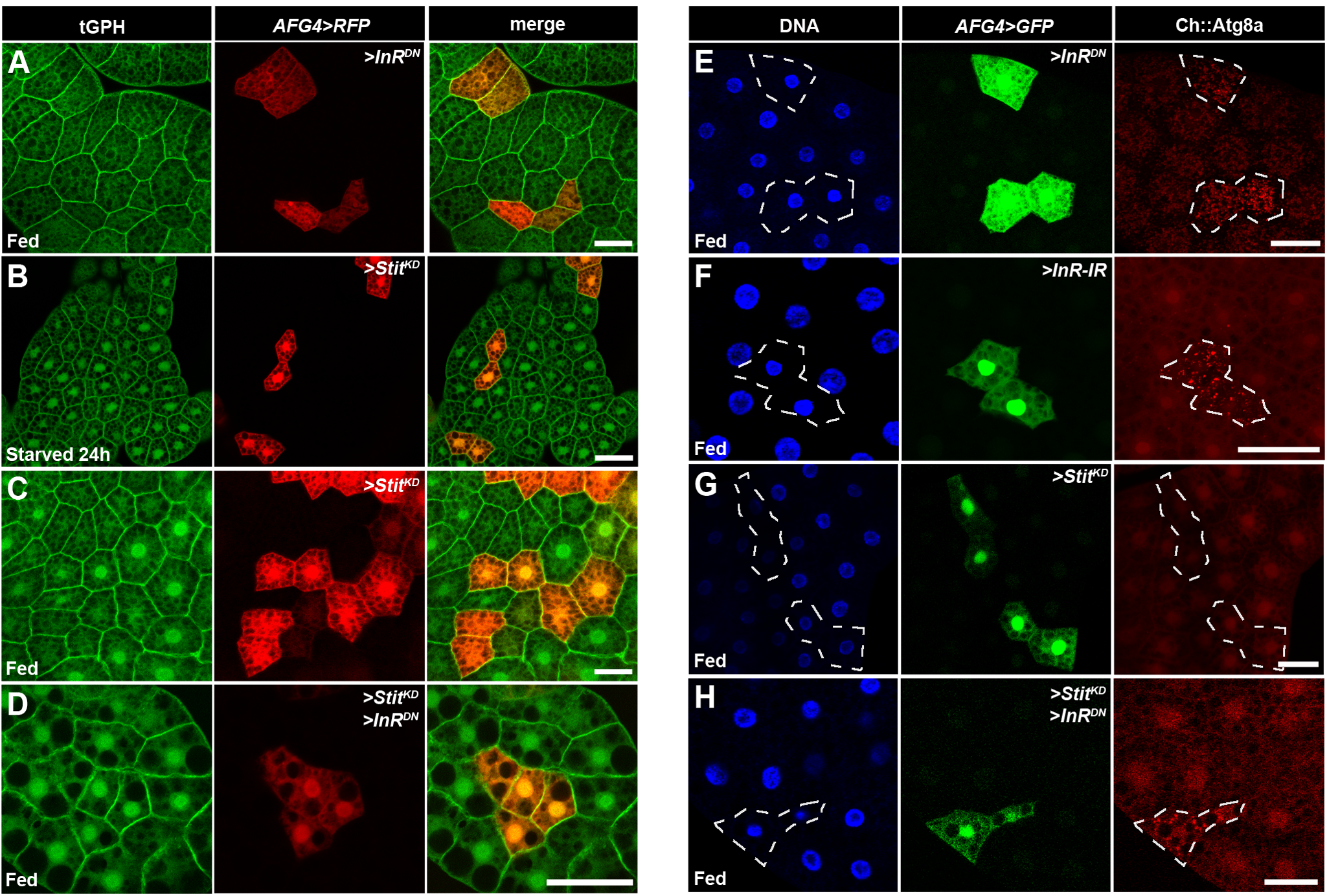

Supplement: Figure S3 — InR but not Stit kinase activity supports growth and suppresses autophagy in the fat body. Inactivation of InR and/or Stit in clones of larval fat body cells (labelled by RFP). (A–D) Clonal disruption of InR (InRDN) or Stit (StitKD), separately or together, had no detectable effect on PI3K-I activity as judged by the tGPH probe (green). (E, F) InR inactivations cause the punctate accumulation of the Ch::Atg8a autophagy reporter in the expressing cells. DNA staining reveals the reduced ploidy/cell size in InRDN- or InR-IR-expressing cells (GFP positive and hatched outline). (G) Fat body clones expressing StitKD (GFP positive and hatched outline) did not induce changes in the accumulation of the autophagy marker in FB cells. (H) Effects of both InR-IR and StitKD expression in clones were comparable to InR-IR alone. Scale bar, 50 µm. (TIF) [file pbio.1001612.s003.tif]

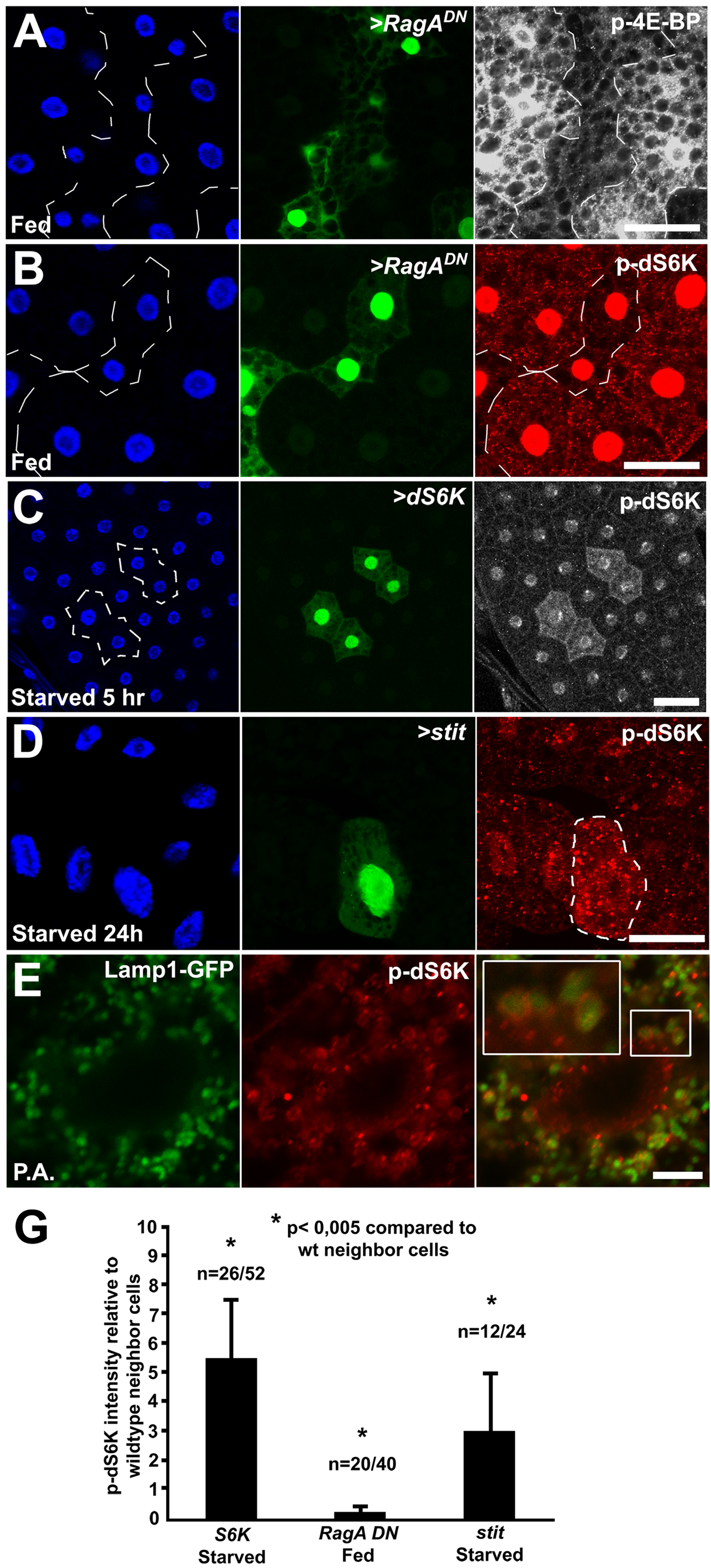

Supplement: Figure S4 — The PI3K-I/TORC1 signaling cassette is required for Stit-dependent protection against starvation. (A) Clones of fat body cells expressing RagADN (marked with GFP in green) showed reduced p-4E-BP levels under fed conditions. (B) Cells expressing RagADN (GFP positive cells outlined with hatched line) showed lower p-dS6K labeling, while wild-type neighbor cells displayed a punctate cytoplasmic labeling pattern. The nuclear p-dS6K labeling was unaltered under these and other conditions (see C and D) and is therefore likely unspecific. Scale bar, 50 µm. (C) Cell clones overexpressing dS6K in larvae under starvation showed increased levels of cytoplasmic p-dS6K signal, while the nuclear labeling remained unaltered. Scale bar, 50 µm. (D) stit expression in cell clones showed increased cytoplasmic accumulation of p-dS6K under starvation conditions. (E) The cytoplasmic puncta of p-dS6K coincided with the late endosomal/lysosomal GFP-tagged protein Lamp1, coating the exterior of the lysosome, in fat body cells undergoing programmed autophagy (P.A.). Inset shows an enlargement of the highlighted area. Scale bar, 10 µm. (G) The intensity of p-dS6K in cells overexpressing dS6K (following 5-h starvation), RagADN, stit (following 24-h starvation), and neighboring wild-type cells was quantified and the ratios were calculated and plotted. The number of overexpressing/neighbor cells (n = overexpressing cells/neighbor cells) quantified is indicated. *indicates significant p values (<0.005) between overexpressing cells and nearest neighbor cells. Error bars indicate standard deviation. (TIF) [file pbio.1001612.s004.tif]

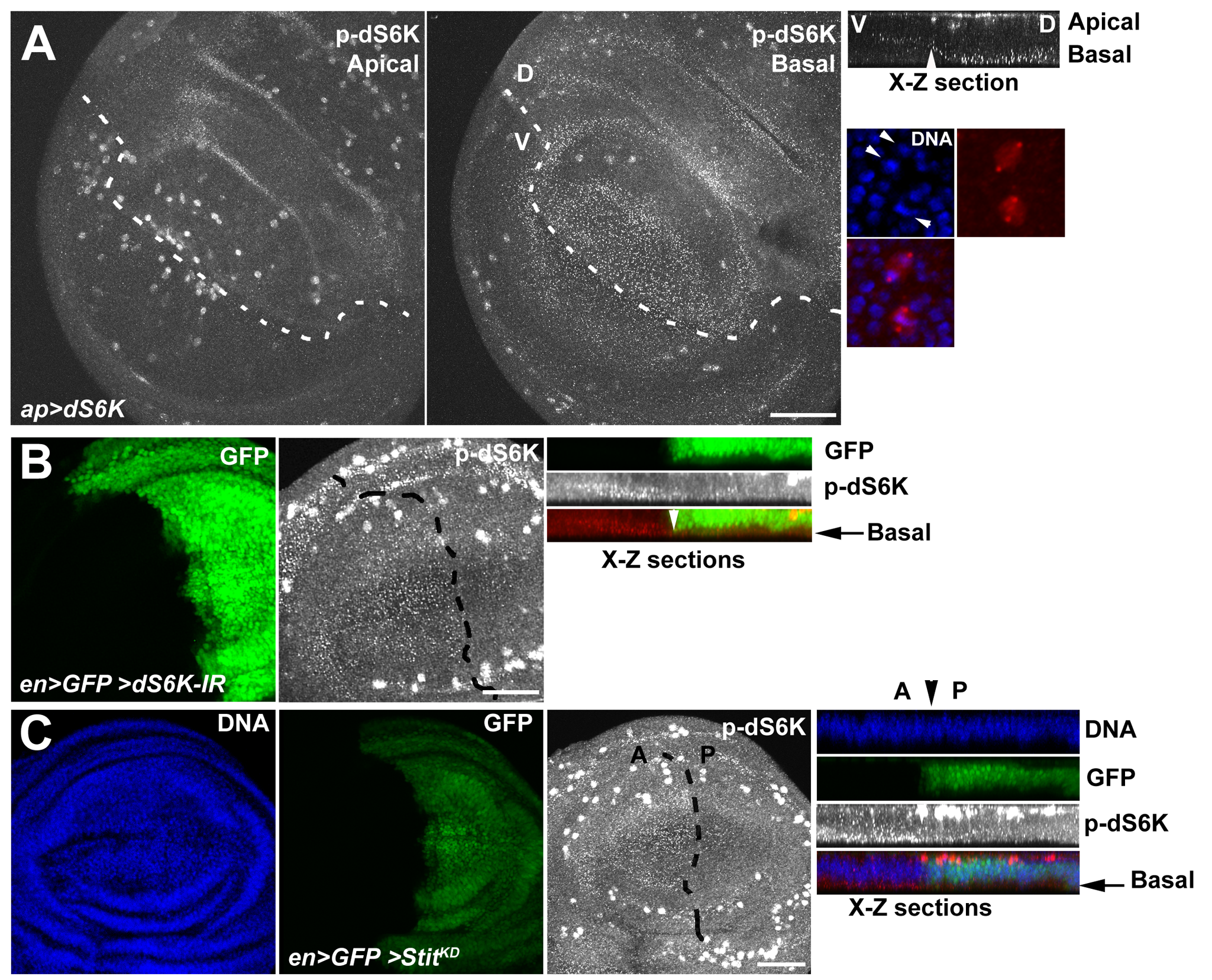

Supplement: Figure S5 — Stit is required for optimal TORC1 signaling in the wing. (A) dS6K overexpression in the dorsal compartment led to a selective basal accumulation of p-dS6K puncta in the dorsal cells of third-instar larval wing discs (right and X–Z section). Dashed line or arrowhead (X–Z section) marks the D/V compartment boundary. Larger clusters of p-dS6K located apically (left) correspond to mitotic cells, magnified in the far right lower panel. Magnified panel measures 25×25 µm. (B) Expression of dS6K-IR in the posterior wing compartment (labelled by GFP) via en-GAL4 lead to a reduction of the basal p-S6K positive puncta, indicating its specificity. The labeling of apical mitotic cells appeared unchanged. Hatching and arrowheads (X–Z section) mark the position of the compartment boundaries in (B) and (C). The basal location of the p-dS6K signal is indicated with a directional arrowhead in X–Z sections. (C) Expression of StitKD in the posterior compartment led to a reduction of the basal p-dS6K signal in the posterior compartment. Scale bar, 50 µm. (TIF) [file pbio.1001612.s005.tif]

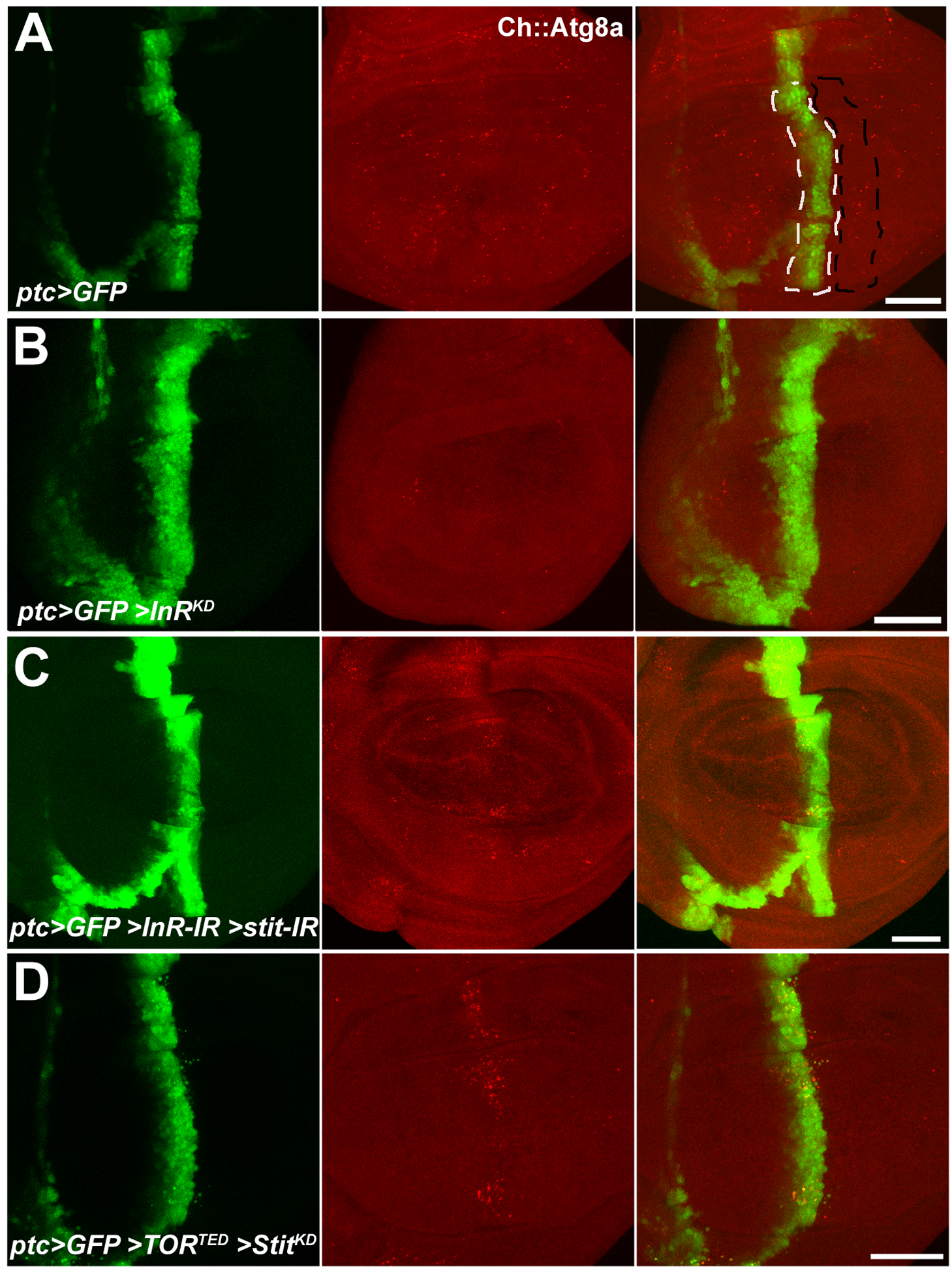

Supplement: Figure S6 — Stit and InR are required to suppress autophagy in the wing. (A) Expression of GFP in the ptc domain of third-instar wing discs did not cause a notable change in the accumulation of Ch::Atg8a expressed under the control of its own promoter. The regions selected for intensity quantification (GFP positive and neighbor region) are indicated. (B) Knock-down of InR (InRDN) in the ptc domain did not change Ch::Atg8a accumulation. (C) Co-expression of InR-IR and stit-IR induced an increase in punctate Ch::Atg8a accumulation. (D) Expression of TORTED together with StitKD did not lead to a further increase in autophagy compared to TORTED alone (Figure 7A). See Figure 7G for quantification. Scale bar, 50 µm. (TIF) [file pbio.1001612.s006.tif]
