# Supplementary material for: Two-Tiered Control of Epithelial Growth and Autophagy by the Insulin Receptor and the Ret-Like Receptor, Stitcher
Source: PLoS Biol. 2013 Jul 23;11(7):e1001612. doi: 10.1371/journal.pbio.1001612 (PMC3720245; doi:10.1371/journal.pbio.1001612)
Supplement: Table S1 — Phenotype strength was graded from severe (++++) to nil (−) unless not determined (ND). Additional phenotypes such as lethality (L) or semilethality (S.L.) were also noted. The percentage flies showing the grade of phenotype is given, as is the number of flies displaying the phenotype when totaling less than 50. Otherwise, greater than 50 flies were examined. Second or third indicates the chromosome the transgene is inserted on when more than one transgene stock was tested. All dS6K transgenes were mutated with different point mutations to be constitutively active. * An extremely small crumpled wing phenotype, while U denotes a bent upward wing and D a bent downward wing. (DOCX) [file pbio.1001612.s007.docx]

**Table S1. Genetic interactions between the Stitcher and Insulin receptor/TOR pathways.**

|  | ***MS1096-GAL4*** | | ***MS1096>stit-IR*** | | ***MS1096>stit*** | | ***ap-GAL4*** | | ***ap>Stit^KD^*** | |
| --- | --- | --- | --- | --- | --- | --- | --- | --- | --- | --- |
| **Transgene** | **Wing bend**  **strength and direction** | **Penetrance**  (% and n) | **Wing bend**  **strength**  **and direction** | **Penetrance**  (% and n) | **Wing phenotype**  **strength and direction** | **Penetrance**  (% and n) | **Wing bend**  **strength**  **And direction** | **Penetrance**  (% and n) | **Wing bend strength and direction** | **Penetrance**  (% and n) |
|  | - | 100% | +++ U | 100% | +++ * | 100% | - | 100% | ++++ U | 100% |
| **>*Rheb* (3^rd^)** | + D | 100% | - | 100% | +++ * | 100% n>20 | +++ D | 100% | + U | 100% |
| **>*Rheb* (2^nd^)** | ++ D | 100% | - | 100% | ++++/L * | L | L | L | L | L |
| **>*Akt*** | - | 100% | + U | 66% | ++++/L * | L | + D | 50% | + U | 75% n>30 |
| **>*dS6K^STQE^*** | + D | 50% | - | 90% n>20 | ++++ * | 100% n>20 | + D | 100% | ++ U | 75% n>20 |
| **>*dS6K^TESA^*** | + D | 10% | + U | 50% | ND |  | ++ D | 75% n>30 | + U | 75% n>30 |
| **>*dS6K^STDETE^*** | + D | 100% | + U | 75% | ND |  | ++ D | S.L. n>10 | ++ U | 75% |
| **>PTEN** | - | 100% | ++ U | 50% | ++ | 100% n>20 | ND |  | ND |  |
| **>*pten-IR*** | - | 100% | ++ U | 100% | ++++/L * | L | - | 100% | ++ U | 100% |
| ***>PI3K* (2^nd^)** | + D | 75% | ++ U | 30% n>30 | ++++/L * | L | ++++ D | S.L. n>10 | ND |  |
| ***>PI3K* (3^rd^)** | - | 100% | +++ U | 100% | ++++/L  * | L | ++ D | 100% n>20 | +++ U | 100% n>20 |
| ***>InR CA*** | + D | 50% | ++ U | 50% | ND |  | + D | 75% n>20 | ++ U | 100% n>20 |
| ***>InR DN*** | ++ U | 100% | ++++ U | 100% | ++ * | 100% | ++ U | 100% n>20 | ++++ U | 100% n>20 |
| ***>TOR^TED^*** | * | 100% | * | 100% | * | 100% | L | L | L | L |
| ***>p35*** | - | 100% | +++ U | 100% |  |  | - | 100% | ++++ U | 100% |
| ***>CycB*** | - | 100% | +++ U | 100% | +++ * | 100% | - | 100% | ++++ U | 100% |
| ***>CycE*** | - | 100% | +++ U | 100% | +++ * | 100% | - | 100% | ++++ U | 100% |
| ***CycE^AR95^/+*** | - | 100% | +++ U | 100% | +++ * | 100% | - | 100% | ++++ U | 100% |
| ***Dap^4^/+*** | - | 100% | +++ U | 100% | +++ * | 100% | - | 100% | ++++ U | 100% |

**Legend Table S1.**

Phenotype strength was graded from severe (++++) to nil (-) unless not determined (ND). Additional phenotypes such as lethality (L) or semi-lethality (S.L.) were also noted. The percentage flies showing the grade of phenotype is given as is the number of flies displaying the phenotype when totaling less than 50. Otherwise greater than 50 flies were examined. 2^nd^ or 3^rd^ indicates the chromosome the transgene is inserted on when more than one transgene stock was tested. All dS6K transgenes were mutated with different point mutations to be constitutively active. * denotes an extremely small crumpled wing phenotype while U denotes a bent upward wing and D a bent downward wing.
